# Study of Cytotoxicity of Spiro-Fused [3-Azabicyclo[3.1.0]hexane]oxindoles and Cyclopropa[a]pyrrolizidine-oxindoles Against Tumor Cell Lines †

**DOI:** 10.3390/ph17121582

**Published:** 2024-11-25

**Authors:** Anton A. Kornev, Stanislav V. Shmakov, Alexander I. Ponyaev, Alexander V. Stepakov, Vitali M. Boitsov

**Affiliations:** 1 Laboratory of Nanobiotechnologies, Saint-Petersburg National Research Academic University of the Russian Academy of Sciences, Saint Petersburg 194021, Russia; 2 Saint-Petersburg State Institute of Technology, Saint Petersburg 190013, Russia; 3 Department of Chemistry, Saint-Petersburg State University, Saint Petersburg 199034, Russia

**Keywords:** 3-spiro[3-azabicyclo[3.1.0]-hexane]oxindole, cyclopropa[a]pyrrolizidine-2,3′-oxindole, antiproliferative activity, cell cycle, morphological changes (cytoskeleton), cell motility, tumor cell lines, 1,3-dipolar cycloaddition, azomethine ylides, cyclopropenes

## Abstract

**Background**: A series of spiro-fused heterocyclic compounds containing cyclopropa[a]pyrrolizidine-2,3′-oxindole and 3-spiro[3-azabicyclo[3.1.0]-hexane]oxindole frameworks were synthesized and studied for their in vitro antiproliferative activity against human erythroleukemia (K562), cervical carcinoma (HeLa), acute T cell leukemia (Jurkat), melanoma (Sk-mel-2) and breast cancer (MCF-7) as well as mouse colon carcinoma (CT26) cell lines. **Methods**: Cell proliferation was evaluated in vitro by MTS assay. Confocal microscopy was used to study actin cytoskeleton structure and cell motility. Cell cycle analysis was evaluated by flow cytometry. **Results**: It was found that compounds **4**, **8**, **18** and **24** showed antiproliferative activity against the Jurkat, K-562, HeLa and Sk-mel-2 cell lines with IC_50_ ranging from 2 to 10 μM (72 h). Evaluation of the impact on cell cycle progression showed that the tested compounds achieved significant cell-cycle perturbation with a higher accumulation of cells in the SubG1 and G0/G1 phases of the cell cycle, in comparison to the negative control. I Incubation with tested compounds led to the disappearance of stress fibers (granular actin was distributed diffusely in the cytoplasm in up to 38% of treated HeLa cells) and changes in the number of filopodia-like deformations (reduced from 93% in control cells to 64% after treatment). The impact on the Sk-mel-2 cell actin cytoskeleton structure was even greater: granular actin was distributed diffusely in the cytoplasm in up to 90% of treated cells while the number of filopodia-like deformations was reduced by up to 23%. A scratch test performed on the human melanoma cell line showed that these cells did not fill the scratched strip and lose their ability to move under treatment. **Conclusions**: The obtained results support the antitumor effect of the tested spiro-compounds and encourage the extension of this study in order to improve their anticancer activity as well as reduce their toxicological risks.

## 1. Introduction

Oncological diseases are one of the most common and lethal diseases in the world and, after cardiovascular diseases, they are the second leading cause of death. The rise of drug resistance and emergence of tumor resistance, along with significant side effects from chemotherapy, diminish the effectiveness of existing anticancer medications and treatments. Although targeted therapies and immunotherapy have shown promising results in specific patients, finding new medications that can help is still a big challenge. Additionally, the emergence of tumor resistance necessitates the creation of anticancer drugs that are not merely variations of traditional drugs but are derived from entirely new compounds. Following these achievements and improvements in cancer understanding, tumor treatment modalities have undergone a remarkable transformation as well [1].

Isolated from microorganisms, natural plant and animal products are prominent sources for anticancer drug research and development [2]. Their enormous number and tremendous structural diversity make them a gift from nature for lead-molecule discoveries. Therefore, natural products and synthetic compounds inspired by them remain outstanding sources for new drug candidates. Many of the anticancer drugs currently in use or undergoing clinical trials are either derived from natural sources or designed based on naturally occurring substances. Most often, these substances are compounds of complex structure that can be produced as a result of multistage synthesis only. Recent progress in complex hetero-system synthesis has sparked considerable interest in creating effective methods for producing various derivatives and structural analogues of these compounds.

Cycloaddition reactions play a pivotal role in organic synthesis by facilitating the formation of cyclic compounds with desirable properties. Among them, 1,3-dipolar cycloaddition reactions have long been recognized as being important for the synthesis of heterocyclic rings. Using new stable or in situ-generated building blocks allows their further modification and diversification. Azomethine ylides are remarkably versatile building blocks in organic synthesis and are known to take part in 1,3-dipolar cycloaddition reactions with a wide range of dipolarophiles. Cycloadditions of azomethine ylides to alkenes and cycloalkenes are well-established reactions in which a wide variety of (poly)hetecyclic scaffolds are formed (including azabicyclo[3.1.0]hexane and azabicyclo[3.3.0]octane scaffolds) [3,4,5,6].

Pyrrolizines and pyrrolizidine alkaloids (both containing a 1-azabicyclo[3.3.0]octane core) as well as their analogues are challenging synthetic targets that are of interest to many medicinal chemists [7,8,9,10] because of their unique structural features and their biological and pharmacological activities (such as antimicrobial [11], antitumor [12,13,14], anti-inflammatory [15,16,17] and anticoagulant (serine protease thrombin inhibitor) effects [18]).

The azabicyclo[3.1.0]hexane moiety is another important scaffold found in many natural products [19,20]; these are used in pharmaceuticals [21,22,23,24] or as valuable intermediate products [25,26,27]. Compounds with azabicyclo[3.1.0]hexane scaffolds show anti-inflammatory [16], antitumor [28], anti-neurodegenerative [29] and antibacterial activity [30,31]; they are antagonists of opioid receptors [23,26] and dopamine D3 receptors [32].

The spiro[pyrrolidine-3,3′-oxindole] moiety is an important scaffold found in a many natural products, pharmaceuticals and biologically active compounds and therefore has served as an inspiration for the development of new therapeutics. This structural fragment is present in oxindole alkaloids characterized by a spiro-fusion of an indole core to a pyrrolidine ring. Such oxindole alkaloids possess a variety of pharmacological properties (cardiovascular [33], neuroprotective [34], antiviral [35] and anticancer [36,37]).

**Figure 1:**
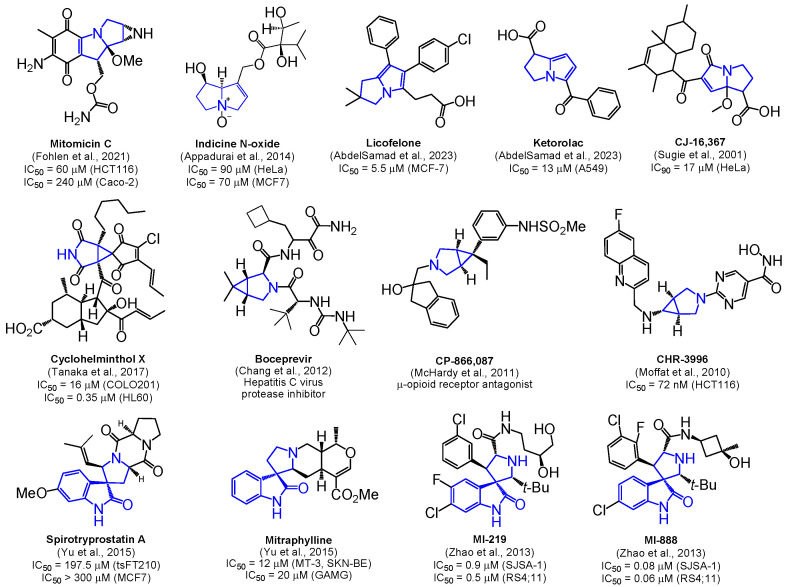
Selected examples of biologically active 1-azabicyclo[3.3.0]octanes (pyrrolizines or pyrrolizidines), 3-azabicyclo[3.1.0]hexanes and spiro[pyrrolidine-3,3′-oxindoles], based on the studies by Fohlen et al. (2021) [10], Appadurai et al. (2014) [12], AbdelSamad et al. (2023) [13], Sugie et al. (2001) [14], Tanaka et al. (2017) [19], Chang et al. (2012) [21], McHardy et al. (2011) [23], Moffat et al. (2010) [28], Yu et al. (2015) [36] and Zhao et al. (2013) [37].

Selected examples of biologically active 1-azabicyclo[3.3.0]octanes (pyrrolizines or pyrrolizidines), 3-azabicyclo[3.1.0]hexanes and spiro[pyrrolidine-3,3′-oxindoles] are presented in Figure 1.

In previous studies by our research group, differently substituted cyclopropenes were widely used as dipolarophiles in 1,3-dipolar cycloaddition reactions generated from the corresponding ketones and *α*-amino acids azomethine ylides. Derivatives of alloxan [38], isatin [39], tryptanthrin [40], ninhydrin [41,42,43,44] and 11*H*-indeno[1,2-*b*]quinoxalin-11-one [45] were used as the ketone components. The products of these reactions were pharmacologically interesting spiro-fused 1-azabicyclo[3.3.0]octanes and 3-azabicyclo[3.1.0]hexanes, some of which were identified as exhibiting in vitro antiproliferative activity [46,47,48].

We here report a study of spiro-fused [3-azabicyclo[3.1.0]hexane]oxindoles and cyclopropa[a]pyrrolizidine-2,3′-oxindoles (readily available via one-pot three-component 1,3-dipolar cycloadditions of various cyclopropenes with azomethine ylides generated in situ from isatins) for their antiproliferative activity against selected tumor cell lines as well as their morphological changes, cell motility and cell cycle perturbations under treatment with the most active products. This article is a revised and expanded version of a paper entitled “Study of cytotoxicity of spiro-fused [3-azabicyclo[3.1.0]hexane]oxindoles against tumor cell lines”, which was presented at 8th International Electronic Conference on Medicinal Chemistry, 1–30 November 2022, MDPI: Basel, Switzerland [48].

## 2. Results and Discussion

### 2.1. Chemistry

Desired spiroadducts were synthesized via three-component one-pot 1,3-dipolar cycloaddition reactions of accordingly substituted cyclopropenes with generated in situ isatin-derived azomethine ylides either as a single (major) isomer or as a mixture of two diastereomeres with up to 85% overall isolation yield (Table 1). The *major* to *minor* ratio in diastereomeric mixtures was found to be within a range of >20–2 to 1, correspondingly. Structures of both diastereomers were assigned on the basis of NMR spectra analysis and unequivocally verified by X-ray crystal analysis (synthesis and structure elucidation was described in detail by us earlier [39,49]).

Despite not all cycloadducts being isolated as single diastereomers and some of them being obtained as an inseparable mixture of two diastereomers, it was decided to study all of them for their in vitro antiproliferative activity against human cancer cell lines. Taking into account that diastereomers may have different activity profiles, mixtures with low activity will not need any further separation trials, while additional steps will be required for those exhibiting considerable activity.

To preliminarily determine whether the synthesized spiro-fused [3-azabicyclo[3.1.0]hexane]oxindoles and cyclopropa[a]pyrrolizidine-2,3′-oxindoles have drug-like properties, an in silico analysis was performed to determine their physicochemical profile using the free online software SwissADME (http://www.swissadme.ch/, accessed on 12 November 2024).

The molecular descriptors were calculated according to Lipinski’s rule of five. According to the rule, orally active drugs should not violate more than one of the following criteria: molecular weight MW < 500 Da, number of hydrogen bond donor HBD < 5, number of hydrogen bond acceptor HBA < 10, number of rotatable bonds nRotB < 10, octanol/water partition coefficient Log P < 5, and topological polar surface area TPSA < 140 Å^2^. The obtained results are presented in Table 2.

### 2.2. Biology

**Figure 2:**
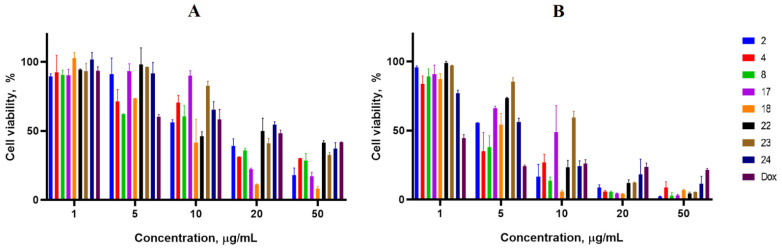
Cytotoxicity of racemic spiro-adducts against the K562 cell line for 24 h (**A**) and 72 h (**B**).

**Figure 3:**
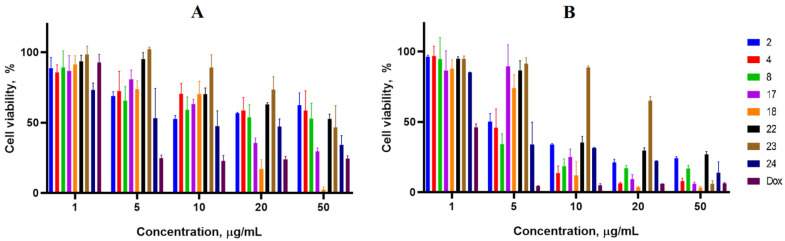
Cytotoxicity of racemic spiro-adducts against the HeLa cell line for 24 h (**A**) and 72 h (**B**).

**Figure 4:**
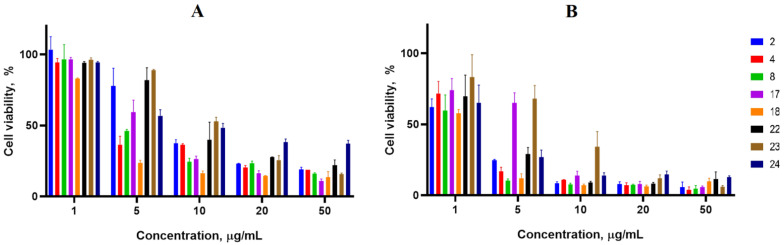
Cytotoxicity of selected racemic spiro-adducts against the Jurkat cell line for 24 h (**A**) and 72 h (**B**).

**Figure 5:**
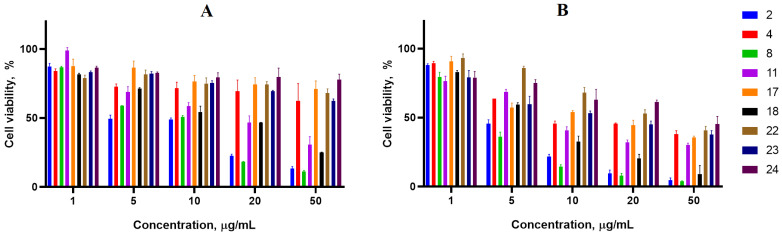
Cytotoxicity of racemic spiro-adducts against the Sk-mel-2 cell line for 24 h (**A**) and 72 h (**B**).

**Figure 6:**
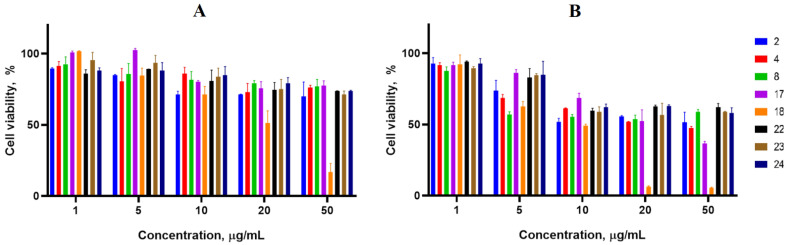
Cytotoxicity of selected racemic spiro-adducts against the MCF-7 cell line for 24 h (**A**) and 72 h (**B**).

**Figure 7:**
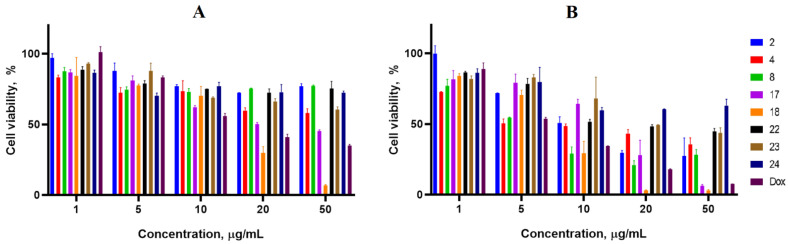
Cytotoxicity of selected racemic spiro-adducts against the CT26 cell line for 24 h (**A**) and 72 h (**B**).

Cancer cell lines are valuable in vitro model systems that are widely used in cancer research and drug discovery. Their use is mainly linked to their possibility to provide for experimental purposes an unlimited source of biological material. Here, the 24 and 72 h in vitro MTS assay was used to determine the antiproliferative activity of synthesized spiro-fused cyclopropa[a]pyrrolizidine-2,3′-oxindoles and [3-azabicyclo[3.1.0]hexane]-oxindoles against human erythroleukemia (K562), acute T cell leukemia (Jurkat), cervical carcinoma (HeLa), melanoma (Sk-mel-2), breast cancer adenocarcinoma (MCF-7) as well as mouse colon carcinoma (CT26) cell lines. It was found that synthesized spiro-fused derivatives significantly reduced the cell proliferation in a time- and concentration-dependent manner. The data for most active compounds are presented in Figure 2, Figure 3, Figure 4, Figure 5, Figure 6 and Figure 7. The full results of these investigations are presented in the Supporting Information Figures S1–S6. Half maximal inhibitory concentration (IC_50_) values of most active spiro-fused adducts against tested cell lines for 24 and 72 h are presented in Table 3. All the tested compounds did not show acute toxicity during the cell proliferation study. An evaluation of the cytotoxicity of the selected compounds towards normal cells (peripheral blood mononuclear cells) was performed additionally to understand their respective relative selectivities. The selectivity index was obtained using the ratio of measured cytotoxicity between the PBMC cells and each of the cancer cell lines, and the results are presented in Table 3.

It is obvious from the obtained data that [3-azabicyclo[3.1.0]hexane]-oxindole derivatives usually show better antiproliferative activity as compared to cyclopropa[a]pyrrolizidine-2,3′-oxindole derivatives. Indeed, among the latter, only adduct **2** exhibited significant antiproliferative activity with IC_50_ ranging from 2 ± 0.2 to 12 ± 2 μg/mL (except for the breast cancer cell line), that equals to 4–23 μM.

The acute T cell leukemia cell line (Jurkat) was the most sensitive to the screened compounds among the tested cancer cell lines with IC_50_ ranging from 1 ± 0.2 to 6 ± 1 μg/mL (72 h), that equals to 4–12 μM.

It was noticed that products with an unsubstituted nitrogen atom in the oxindol unit were more active against all studied cell lines. Indeed, adducts bearing methyl, phenyl or benzyl substituents fitted to oxindole nitrogen exhibited less activity. At the same time, among the tested products, those bearing three phenyl substituents at the cyclopropane unit were usually more effective. All the active compounds significantly reduced proliferative activity at concentrations less than 10 μg/mL. Since cycloadducts **2**, **4**, **22** and **23** consist of diastereomer mixtures at a range of 5–2 to 1, additional attempts are required for their separation and isolation as single isomers. If it is assumed that other isomers exhibit no activity, this can lead to a 2–5-fold activity increase.

Based on the obtained data, compounds that showed better antiproliferative activity were selected for further evaluation of their impact on cell motility, cytoskeletal morphology and progression of the cell cycle.

### 2.3. Actin Cytoskeleton Changes

It is known that actin plays an important role in vital cellular processes, providing a number of functions such as cell adhesion, migration and morphogenesis [50,51]. The actin cytoskeleton may serve as an additional target of antitumor chemotherapy [52,53]. Tumor transformation causes reorganization of the actin cytoskeleton, leading to changes in cell motility. A correlation between increased tumor cell migration activity and actin assembly and organization was observed [54,55]. Actin organization structural features can serve as criteria for assessing metastasis potential [56]. The HeLa cell line is extensively used to analyze actin cytoskeleton structure and is characterized by the presence of filopodia and actin stress fibers [57,58].

The structure of the actin cytoskeleton of HeLa and Sk-mel-2 cells was assessed after the impact of the most active spiro-fused oxindole derivatives by the availability of stress fibers and the presence of filopodia-like protrusions.

**Figure 8:**
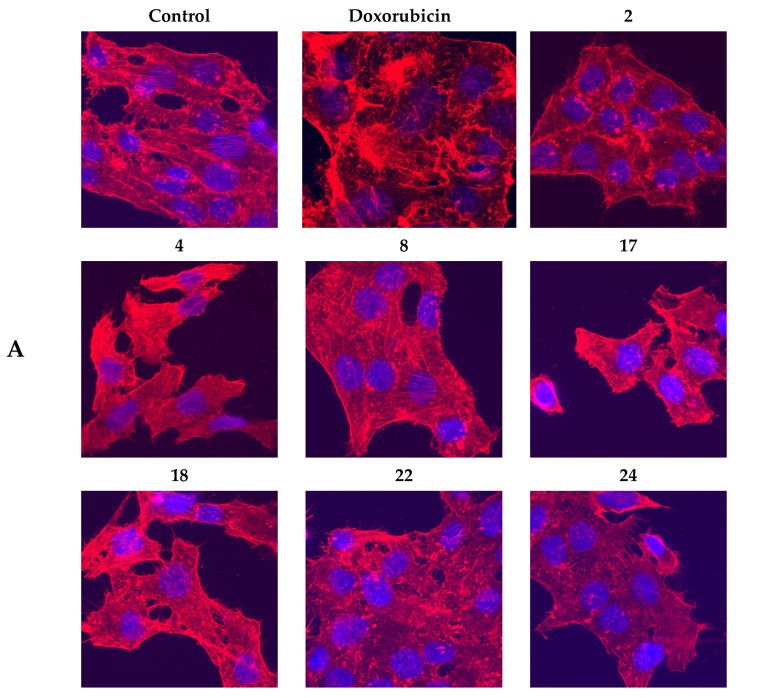

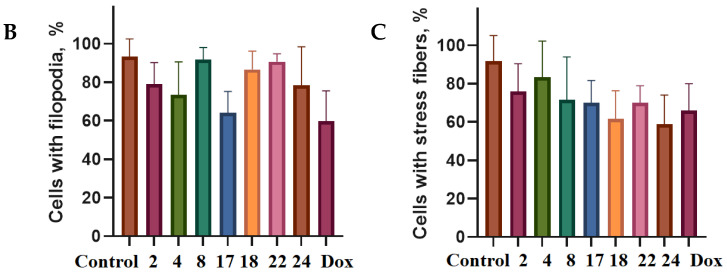
State of actin cytoskeleton of HeLa cells after treatment with compounds **2**, **4**, **8**, **17**, **18**, **22** and **24** (10 μg/mL). (**A**) Images demonstrating the different stages of the cell actin cytoskeleton. (**B**) Histograms demonstrating the percentage of cells with filopodia-like deformations. (**C**) Histograms demonstrating the percentage of cells with normal stress fibers.

**Figure 9:**
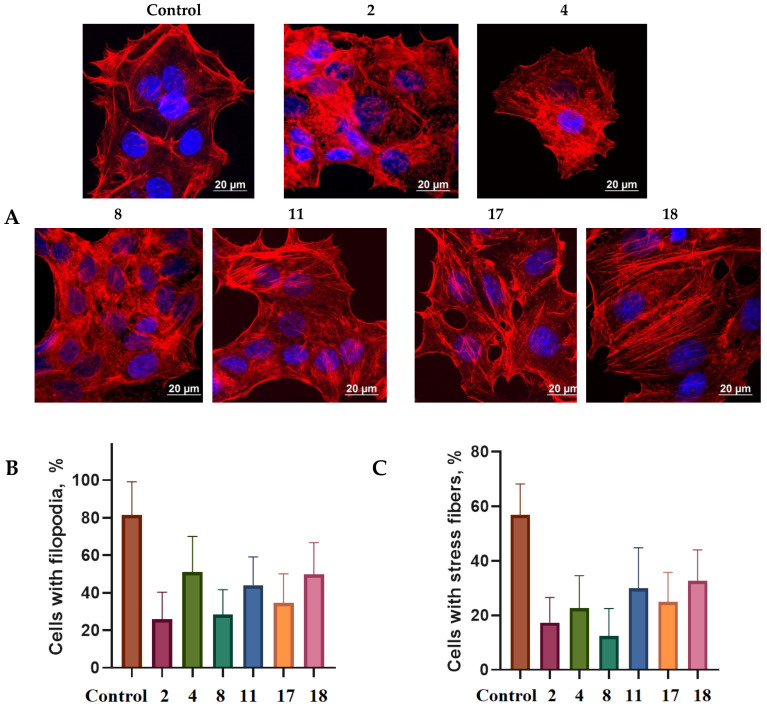
State of actin cytoskeleton of Sk-mel-2 cells after treatment with compounds **2**, **4**, **8**, **11**, **17** and **18** (10 μg/mL). (**A**) Images demonstrating the different stages of the cell actin cytoskeleton. (**B**) Histograms demonstrating the percentage of cells with filopodia-like deformations. (**C**) Histograms demonstrating the percentage of cells with normal stress fibers.

It was found using confocal microscopy that incubation with spiro-fused [3-azabicyclo[3.1.0]hexane]oxindoles **4**, **8**, **17**, **18**, **22**, **24** and cyclopropa[a]pyrrolizidine-2,3′-oxindole **2** led to significant changes in the HeLa cells’ actin cytoskeleton structure leading to the disappearance of stress fibers (granular actin was distributed diffusely in the cytoplasm in up to 38% of treated cells) and changes in the number of filopodia-like deformations (reduced from 93% in control cells to 64% after treatment). The impact on the Sk-mel-2 cells’ actin cytoskeleton structure was even greater: granular actin was distributed diffusely in the cytoplasm in up to 90% of treated cells while the number of filopodia-like deformations was reduced by up to 23%. Such changes in the cytoskeleton may indicate a change in the motor activity of cells, which may indicate a decrease in the ability of tumor cells to metastasize. At the same time, experimental effects did not cause fragmentation of the nucleus, which indicates the absence of pro-apoptotic activity. Data on actin cytoskeleton structure as well as histograms demonstrating the percentage of cells with filopodia-like deformations and disassembled stress fibers are combined in Figure 8 and Figure 9.

### 2.4. Inhibition of Cell Motility Evaluated by Scratch Test

Cell motility is a basic and ancient cellular behavior that is caused by cell invasion and metastasis [59]. Metastasis is one of the main causes of mortality of cancer patients that accounts for about 90% of cancer-related deaths. The process of metastasis is the migration of cancer cells from their originating site to distant organs [60]. A major challenge in understanding metastatic tumor spread in patients is that the process cannot be observed or manipulated directly. The scratch test is a simple model used to assess the impact of different effects on cell motility and metastasis.

**Figure 10:**
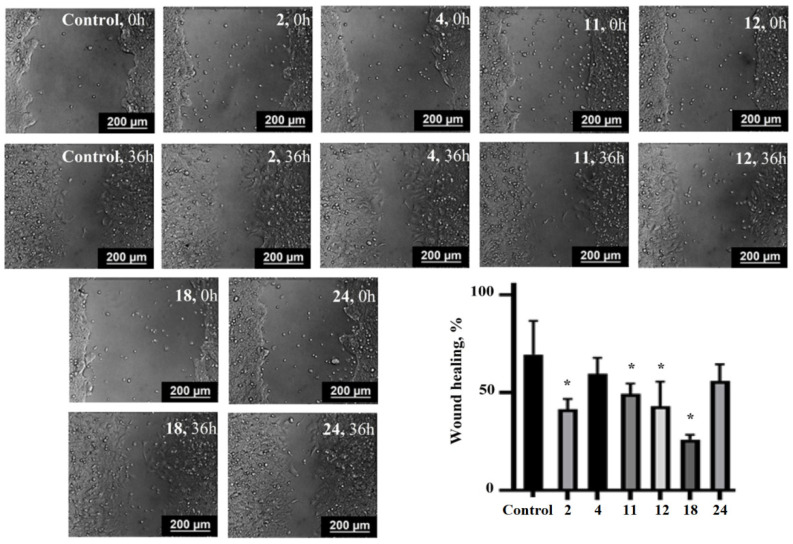
Microscopic images of the Sk-mel-2 cell wound area in the scratch assay and wound area (%) in the scratch assay after 36 h incubation with spiro-fused [3-azabicyclo[3.1.0]hexane]oxindoles **4**, **11**, **12**, **18**, **24** and cyclopropa[a]pyrrolizidine-2,3′-oxindole **2** (10 μg/mL). * *p* value < 0.05.

To assess the potential ability of spiro-fused [3-azabicyclo[3.1.0]hexane]oxindoles **4**, **11**, **12**, **18**, **24** and cyclopropa[a]pyrrolizidine-2,3′-oxindole **2** to inhibit metastasis associated with cell motility, a scratch test was performed on the human melanoma (Sk-mel-2) cell line. Different fields were analyzed by bright field, and each scratch area was photographed at 0 and 36 h for non-toxic and fast cell visualization. The results are shown in Figure 10. Nontreated Sk-mel-2 cells filled 69.5 ± 17.2, 41.5 ± 5.3, 59.7 ± 8.1, 49.3 ± 5.3, 43.0 ± 12.6, 26.1 ± 2.5 and 55.9 ± 8.6% of the the scratched strip while under treatment with spiro-fused oxindole derivatives **2**, **4**, **11**, **12**, **18** and **24** cells, respectively. Therefore, treated Sk-mel-2 cells lost their ability to move and did not fill the scratched strip; however their structure–activity relationship needs further evaluation. The presented results indicate that the tested compounds can block the cellular movement of tumor cells.

### 2.5. Cell Cycle Analysis

Since growth inhibition may be a result of cell cycle arrest at a particular point, the specific antiproliferative activity of several cycloadducts was further evaluated by flow cytometry to study their impact on the progression of the cell cycle in K562 cells. The assay utilizes propidium iodide-based staining of DNA content to distinguish and measure the accumulation of cells in each cell cycle phase (SubG, G0/G1, S and G2/M).

**Figure 11:**
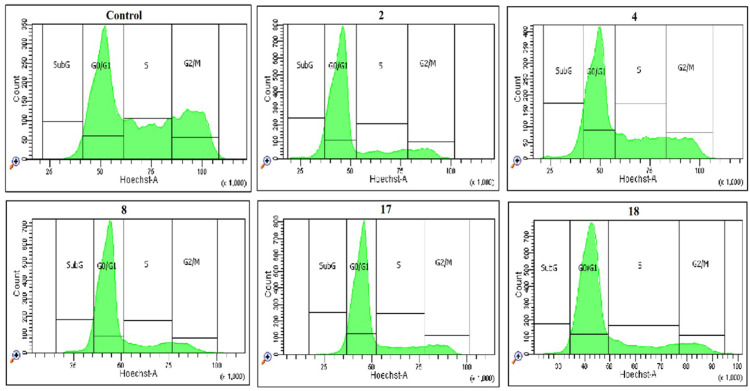
The effect of cycloadducts **2**, **4**, **8**, **17**, **18** at a concentration of 10 μg/mL on the distribution of **K562** cells in the cell cycle.

Analysis of the experimental results showed that screened spiro-fused [3-azabicyclo[3.1.0]hexane]oxindole derivatives **4**, **8**, **17**, **18** and cyclopropa[a]pyrrolizidine-2,3′-oxindole **2** stopped the K562 cell cycle in SubG1 and G0/G1 phases, in comparison to the negative control (Figure 11, Table 4). Thus, after 24 h incubation with the tested compounds at 10 μg/mL concentration, the accumulation of cells in the G0/G1 phase of the cycle increased from 40.5% (control sample) to 73.5% for compound **8**. The accumulation of cells in the SubG1 phase of the cycle increased from 4.5% (control sample) to 7.7% after incubation with compound **2**. The total percentage of cells in the SubG1 and G0/G1 phases increased from 45.0% to 74.0–77.5% after incubation with compounds **2**, **8**, **17** and **18**. The accumulation of cells in the synthetic phase (S) of the cycle was also lower for treated cells (up to 14.3–19.0%) than the control sample (31.9%). The accumulation of cells in the G2/M phase of the cycle decreased from 23.1% for the control sample to 5.9–8.8% for those treated with compounds **2**, **8**, **17** and **18**.

Additionally, the concentration-dependent effects of spiro-fused [3-azabicyclo[3.1.0]hexane]oxindole derivatives **4**, **8**, **17**, **18** and cyclopropa[a]pyrrolizidine-2,3′-oxindole **2** on cell cycle phase distribution was evaluated. The results of this investigation are summarized in Table 4 and Figure S7. Analysis of the experimental results showed that, generally, a 24 h incubation of K562 cells with the screened compounds at concentrations of 5, 10 and 20 μg/mL led to further increased SubG1 and G0/G1 populations and decreased S- and G2/M populations.

These findings indicate that the tested compounds prevent cancer cells from starting DNA division.

## 3. Materials and Methods

### 3.1. Chemistry

^1^H (400 MHz) and ^13^C (101 MHz) NMR spectra were recorded with a Bruker Avance 400 spectrometer (Bruker Biospin, Rheinstetten, Germany). Chemical shifts are reported in ppm relative to solvent residual signals (7.26 and 77.17 ppm for ^1^H and ^13^C in CHCl_3_; 2.50 and 39.52 ppm for ^1^H and ^13^C in DMSO-*d*_5_) as internal standards. Melting points were determined using a Boetius instrument. Cyclopropenes were prepared according to the literature data [61,62,63], while α-amino acids and isatins were obtained from commercial sources. Reaction course, purity and individuality of the compounds were monitored by TLC on Silufol UV-254 plates. Preparative TLC was performed on a 5–40 mesh silica gel, eluting with a petroleum ether–ethyl acetate mixture.

The general procedure for the three-component one-pot cycloaddition reaction of isatins, *α*-amino acids and cyclopropenes was as follows: A mixture of cyclopropene (0.4 mmol), isatin (0.4 mmol) and *α*-amino acid (0.6 mmol) was refluxed in methanol (8 mL) for 4 h under a nitrogen atmosphere. Reaction progress was monitored by TLC. After completion of the reaction, the solvent was removed under reduced pressure. The residue was recrystallized or subjected to silica gel PTLC to obtain the pure products. The ^1^H NMR spectra of compounds 1–29 are presented in Figures S8–S36. Spectral and physical data for all the obtained products were identical to those described by us earlier for compounds 1–25 at [39]; for others, see [49].

#### In Silico Analysis

The molecular descriptors of the synthesized spiro-fused [3-azabicyclo[3.1.0]hexane]oxindoles and cyclopropa[a]pyrrolizidine-2,3′-oxindoles were determined with the widely used free online software SwissADME (http://www.swissadme.ch/, accessed on 12 November 2024) and analyzed according to Lipinski’s rule of five. In all the cases, the descriptors calculation was conducted by uploading the standard smile files onto the web server.

### 3.2. Cell Culture and Culturing Conditions

The human erythroleukemia (K-562), cervical carcinoma (HeLa) as well as mouse colon carcinoma (CT26) cell lines were obtained from the Bank of Cell Cultures of the Institute of Cytology of the Russian Academy of Sciences; the human melanoma (Sk-mel-2) cell line was obtained from the Bank of Cell Cultures of the Institute of Cytology and Genetics, Siberian Branch of the Russian Academy of Sciences. The acute T cell leukemia (Jurkat) cell line was obtained from the Bank of Cell Cultures of the Institute of Immunology of the Russian Academy of Medical Sciences. The peripheral blood mononuclear cells (PBMC) were isolated from the blood of healthy donors. K-562, Jurkat, MCF-7, PBMC and CT26 cells were grown on RPMI medium (Hyclone, GE Healthcare Life Sciences, Logan, UT, USA) supplemented with 10% (*v*/*v*) fetal bovine serum (Hyclone, GE Healthcare Life Sciences, Logan, UT, USA) and gentamicin (Sigma-Aldrich, St. Louis, MO, USA) at 37 °C in a humidified atmosphere with 5% CO_2_. HeLa and Sk-mel-2 cells were cultured with the same supplements and conditions in DMEM medium (Hyclone, GE Healthcare Life Sciences, Logan, UT, USA).

### 3.3. Cell Proliferation Assay

To evaluate the in vitro toxicity of the compounds synthesized, cells were seeded into 96-well plates at a density of 5 × 10^3^ cells per well. On the next day, the tested compounds were added to the wells at concentrations ranging from 1 to 30 μg/mL, followed by incubation for 1 and 3 days. Cell proliferation was determined by adding 20 μL of MTS reagent (BioVision, Milpitas, CA, USA) stock solution per well. The plates were incubated for 2 h at 37 °C in a humidified, 5% CO_2_ atmosphere. The plates were then read at 495 nm using a plate spectrophotometer (Multiskan GO, Thermo Fisher Scientific, Waltham, MA, USA). All samples were measured in triplicate.

### 3.4. Actin Cytoskeleton Staining

HeLa cells were seeded at a density of 2 × 10^5^ cells per dish onto a petri dish with cover slips and incubated for 24 h. After that, cells were treated with chosen compounds (10 μg/mL) for 24 h. The medium was removed and cells were fixed with 4% paraformaldehyde (Sigma-Aldrich, St. Louis, MO, USA), washed three times with PBS, and permeabilized with 0.3% Triton-X100 (Sigma-Aldrich, St. Louis, MO, USA). The cells were rinsed three times with PBS. Actin filaments (microfilaments) were stained with rhodamine–phalloidin (Invitrogen, Carlsbad, CA, USA) at 37 °C for 15 min. The samples were rinsed three times with PBS, followed by embedding in Fluoroshield medium (Sigma-Aldrich, St. Louis, MO, USA). Cells were imaged using an Axio Observer Z1 confocal microscope (Carl Zeiss MicroImaging GmbH, Jena, Germany). In each experiment, at least 30 cells were imaged. Images were analyzed by a pathologist blinded to the treatment mode used for each group using ImageJ software, 1.52v.

### 3.5. Evaluation of Cell Motility by Scratch Test

Cells were seeded at a density of 5 × 10^5^ cells per dish onto petri dishes and grown to confluency. A 200 μL pipette tip was used to make scratch wounds and detached cells were removed after that by washing with phosphate-buffered saline. Culture media was replaced with serum-free DMEM in order to inhibit cell proliferation. Compounds to be screened were added at a dose of 10 μg/mL to the cultures and incubated for 36 h. Different fields were analyzed by bright field, and each scratch area was photographed at 0 and 36 h. Images were captured using an Axio Observer Z1 confocal microscope (Carl Zeiss MicroImaging GmbH, Jena, Germany). The percent of wound closure in five randomly chosen fields was calculated with ImageJ software, 1.52v.

### 3.6. Cell Distribution over the Different Phases of the Cell Cycle

The distribution of K562 cells in the G0/G1-, S- and G2/M-phases of the cell cycle was obtained by quantification of DNA content using flow cytometry in DAPI-stained cells. Briefly, cells were seeded at a density of 5 × 10^4^ cells per well in 24-well plates. After a 24 h incubation, cells were exposed to 5, 10 or 20 μg/mL of the screened compounds for another 24 h. After incubation with drugs, the cells were harvested by pipetting. This was followed by treatment with 0.2 mg/mL saponin (Fluka, Waltham, MA, USA) and 0.05 mg/mL DAPI (Thermo scientific, Rockford, IL, USA). After washing, the samples were analyzed using a standard flow cytometer (BD FACSCanto II, Becton Dickinson, San Jose, CA, USA). A total of 10,000 events were acquired for the samples. Data processing was performed using the BD FACSDiva 9.0 software.

### 3.7. Statistical Analysis

Statistical processing of results was performed using Statistica 6.0. All data from the three independent experiments were used for measuring the means ± standard deviations (mean ± SD), which were compared using the Student’s *t*-test or a nonparametric Wilcoxon Mann–Whitney U test.

## 4. Conclusions

A series of heterocyclic compounds containing 3-spiro[3-azabicyclo[3.1.0]hexane]oxindole and cyclopropa[a]pyrrolizidine-2,3′-oxindole frameworks were studied for their antiproliferative activity against the human K562, Jurkat, HeLa, Sk-mel-2, MCF-7 as well as mouse CT26 cell lines. Derivatives with the [3-azabicyclo[3.1.0]hexane]-oxindole moiety showed better antiproliferative activity as compared to cyclopropa[a]pyrrolezidine-2,3′-oxindoles. It was found that the Jurkat cell line was the most sensitive to the screened compounds among the tested cell lines, with IC_50_ ranging from 2 ± 1 to 12 ± 1 μM (for compounds **2**, **4**, **8**, **17**, **18**, **22**, **23** and **24** after 72 h treatment). It was noticed that products with an unsubstituted nitrogen atom in the oxindol unit were more active against all studied cell lines. At the same time, those products bearing three phenyl substituents at the cyclopropane unit were usually more effective. All the active compounds significantly reduced proliferative activity at concentrations less than 12 μM. It was found that compounds **4**, **8**, **18** and **24** showed antiproliferative activity against the Jurkat, K-562, HeLa and Sk-mel-2 cell lines with IC_50_ ranging from 2 to 10 μM after 72 h of treatment. In agreement with the DNA cytometry studies, the tested compounds achieved significant cell-cycle perturbation with a higher accumulation of cells in SubG1 and G0/G1 phases of up to 74.0–77.5% (as compared to 45.0% in the control sample). In agreement with confocal microscopy studies, actin filaments disappeared and granular actin was distributed diffusely in the cytoplasm of up to 38% of treated HeLa cells. Additionally, the number of HeLa cells with filopodium-like membrane protrusions was significantly reduced after treatment with some of the tested compounds (from 93% in control cells to 64% after treatment). The impact on Sk-mel-2 cells’ actin cytoskeleton structure was even greater: granular actin was distributed diffusely in the cytoplasm in up to 90% of treated cells while the number of filopodia-like deformations was reduced by up to 23%. These results indirectly suggest a decrease in cell motility. A scratch test performed on the human melanoma cell line showed that these cells did not fill the scratched strip and lost their ability to move under treatment. The obtained results support the antitumor effect of the studied compounds and encourage the extension of this study in order to improve the anticancer activity and reduce the toxicological risks of the obtained compounds.

## Figures and Tables

**Table 1:** Synthesis of spiro-adducts via three-component one-pot cycloaddition reactions of cyclopropenes, isatines and *α*-amino acids ^a,b,c^.

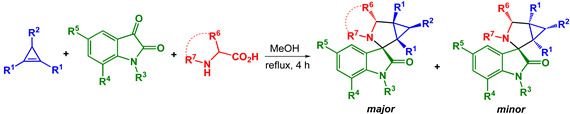
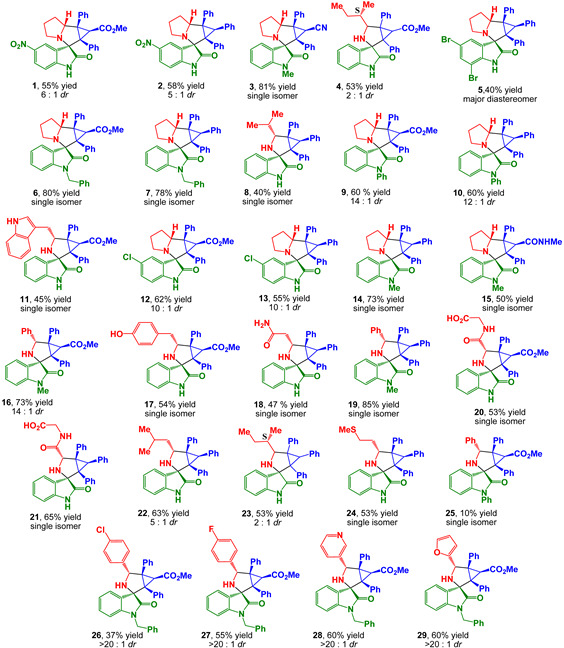

^a^ Reactions of cyclopropene (0.4 mmol), isatine (0.4 mmol) and *α*-amino acid (0.6 mmol) were carried out in methanol (8 mL) under reflux for 4 h under a nitrogen atmosphere. ^b^ Isolated yield. ^c^ The *dr* values were determined by ^1^H NMR of the crude mixture.

**Table 2:** Physicochemical profiles of compounds according to Lipinski’s rule of five.

Compound	MW	nHBD	nHBA	Log P	nRotB	TPSA, Å^2^	N_Violation_	Meets Lipinski’s Criteria
	<500	<5	<10	≤5	<10	<140	<1	Yes/No
**1**	495.53	1	6	3.04	5	104.46	0	Yes
**2**	513.59	1	4	4.45	4	78.16	2	No
**3**	431.53	0	3	3.92	2	47.34	0	Yes
**4**	466.57	2	4	4.30	6	67.43	0	Yes
**5**	626.38	1	2	6.41	3	32.34	2	No
**6**	540.65	0	4	5.11	6	49.85	2	No
**7**	558.71	0	2	6.54	5	23.55	2	No
**8**	470.60	2	2	5.46	4	41.13	1	Yes
**9**	526.62	0	4	5.13	5	49.85	2	No
**10**	544.68	0	2	6.54	4	23.55	2	No
**11**	539.62	3	4	4.66	6	83.22	1	Yes
**12**	484.97	1	4	4.31	4	58.64	1	Yes
**13**	503.03	1	2	5.75	3	32.34	2	No
**14**	482.61	0	2	5.38	3	23.55	1	Yes
**15**	463.57	1	3	3.53	4	52.65	0	Yes
**16**	500.59	1	4	4.45	5	58.64	2	No
**17**	516.59	3	5	4.15	6	87.66	1	Yes
**18**	485.58	3	3	7.76	5	84.22	0	Yes
**19**	518.65	1	2	5.91	4	32.34	2	No
**20**	511.53	4	7	1.50	8	133.83	1	Yes
**21**	529.59	4	5	2.90	7	107.53	1	Yes
**22**	484.63	2	2	5.77	5	41.13	1	Yes
**23**	484.63	2	2	5.76	5	41.13	1	Yes
**24**	502.67	2	2	5.51	6	6.43	2	No
**25**	562.66	1	4	5.57	6	58.64	2	No
**26**	611.13	1	4	6.10	7	58.64	2	No
**27**	594.67	1	5	5.91	7	58.64	2	No
**28**	577.67	1	5	4.89	7	71.53	2	No
**29**	566.65	1	5	4.98	7	71.78	2	No

MW: molecular weight; nHBD: number of hydrogen bond donors; nHBA: number of hydrogen bond acceptors; Log P: logarithm of partition coefficient of the compound between n-octanol and water; nRotB: number of rotatable bonds; TPSA: topological polar surface area; N_Violation_: number of violated criteria.

**Table 3:** IC_50_ values of most active spiro-fused [3-azabicyclo[3.1.0]hexane]oxindoles against the **K562**, **HeLa**, **Jurkat**, **CT26** and **PBMC** cell lines for 24 and 72 h.

Compound	IC_50_, μM																			
	K562			HeLa			Jurkat			MCF-7			Sk-mel-2			CT26			PBMC	
	24 h	72 h	SI	24 h	72 h	SI	24 h	72 h	SI	24 h	72 h	SI	24 h	72 h	SI	24 h	72 h	SI	24 h	72 h
**2**	29 ± 2	8 ± 1	11	>40	12 ± 1	7	18 ± 2	4 ± 1	22	>40	>40	2	14 ± 1	12 ± 1	7	>40	23 ± 2	4	121 ± 8	84 ± 8
**4**	32 ± 2	6 ± 1	14	>40	9 ± 1	11	11 ± 1	4 ± 1	22	>40	>40	2	>40	9 ± 1	11	>40	21 ± 2	4	174 ± 17	92 ± 9
**8**	25 ± 1	6 ± 1	7	>40	8 ± 1	6	11 ± 1	2 ± 1	22	>40	>40	1	15 ± 1	8 ± 1	6	>40	11 ± 1	4	83 ± 7	47 ± 5
**11**	7 ± 1	7 ± 1	– *	15 ± 2	22 ± 2	– *	– *	– *	– *	– *	– *	– *	33 ± 3	15 ± 1	– *	>40	19 ± 2	– *	– *	– *
**17**	35 ± 5	14 ± 1	8	31 ± 2	14 ± 2	8	12 ± 1	8 ± 1	15	>40	>40	2	>40	14 ± 2	8	>40	23 ± 2	5	127 ± 16	112 ± 9
**18**	14 ± 2	6 ± 1	2	23 ± 2	10 ± 2	1	6 ± 1	2 ± 1	6	>40	14 ± 1	1	29 ± 2	10 ± 1	1	27 ± 2	10 ± 1	1	29 ± 3	12 ± 1
**22**	>40	12 ± 1	5	>40	23 ± 2	3	21 ± 2	4 ± 1	16	>40	>40	1	>40	23 ± 2	3	>40	39 ± 3	2	132 ± 16	64 ± 6
**23**	>40	21 ± 2	8	>40	>40	3	25 ± 2	12 ± 1	13	>40	>40	3	>40	>40	3	>40	>40	3	177 ± 20	165 ± 16
**24**	>40	10 ± 1	9	20 ± 2	8 ± 1	12	22 ± 2	4 ± 1	23	>40	>40	1	>40	8 ± 1	12	>40	>40	1	113 ± 14	92 ± 11
Doxorubicin	16 ± 3	1 ± 0.4	9	4 ± 1	1 ± 0.1	9	– *	– *	– *	– *	– *	– *	– *	– *	– *	19 ± 3	6 ± 1	2	11 ± 1	9 ± 1

*—not determined; SI—selectivity index, calculated at 72 h.

**Table 4:** The effect of cycloadducts **2**, **4**, **8**, **17**, **18** at concentrations of 5, 10 and 20 μg/mL on the distribution of **K562** cells in the cell cycle.

Sample	SubG1	G0/G1	S	G2/M		Sample	SubG1	G0/G1	S	G2/M
**Control**	4.5 ± 0.9	40.5 ± 1.3	31.9 ± 1.0	23.1 ± 1.7		**8 (5 mg/mL)**	2.1 ± 0.1	63.1 ± 0.4	25.7 ± 0.1	9.2 ± 0.3
**Doxorubicin (1 μg/mL)**	43.0 ± 1.0	45.6 ± 0.8	8.8 ± 1.7	2.2 ± 0.3		**8 (10 μg/mL)**	4.1 ± 0.2	73.5 ± 0.5	16.6 ± 0.2	5.9 ± 0.2
**Doxorubicin (5 μg/mL)**	93.1 ± 0.7	5.9 ± 0.6	0.8 ± 0.1	0.2 ± 0.1		**8 (20 μg/mL)**	3.6 ± 0.2	72.4 ± 0.2	17.0 ± 0.2	7.1 ± 0.1
**2 (5 μg/mL)**	5.3 ± 0.3	47.7 ± 0.5	29.5 ± 0.6	17.4 ± 0.4		**17 (5 μg/mL)**	2.5 ± 0.3	55.6 ± 0.3	25.1 ± 0.4	16.7 ± 1.0
**2 (10 μg/mL)**	7.7 ± 0.4	68.8 ± 0.5	15.6 ± 0.4	7.8 ± 0.3		**17 (10 μg/mL)**	5.3 ± 0.4	71.5 ± 0.5	14.3 ± 0.1	8.8 ± 0.4
**2 (20 μg/mL)**	5.8 ± 0.3	72.5 ± 0.5	14.5 ± 0.2	7.3 ± 0.2		**17 (20 μg/mL)**	1.8 ± 0.2	74.5 ± 0.8	16.4 ± 0.5	7.2 ± 0.3
**4 (5 μg/mL)**	4.3 ± 0.2	52.6 ± 0.7	26.6 ± 0.7	16.5 ± 0.2		**18 (5 μg/mL)**	3.2 ± 0.1	51.0 ± 0.7	31.4 ± 0.1	14.4 ± 0.5
**4 (10 μg/mL)**	4.8 ± 0.5	56.9 ± 0.6	26.4 ± 0.3	11.9 ± 0.6		**18 (10 μg/mL)**	3.9 ± 0.2	70.1 ± 0.5	19.0 ± 0.6	7.0 ± 0.4
**4 (20 μg/mL)**	4.9 ± 0.2	71.2 ± 0.4	17.4 ± 0.4	6.9 ± 0.4		**18 (20 μg/mL)**	3.6 ± 0.1	65.0 ± 0.4	22.8 ± 0.4	8.6 ± 0.3

## Data Availability

The data presented in this study are available on reasonable request from the corresponding authors.

## Supplementary Materials

The following supporting information can be downloaded at: https://www.mdpi.com/article/10.3390/ph17121582/s1.
