# Supplementary material for: Study of Cytotoxicity of Spiro-Fused [3-Azabicyclo[3.1.0]hexane]oxindoles and Cyclopropa[a]pyrrolizidine-oxindoles Against Tumor Cell Lines
Source: Pharmaceuticals (Basel). 2024 Nov 25;17(12):1582. doi: 10.3390/ph17121582 (PMC11680018; doi:10.3390/ph17121582)
Supplement: Supplementary file 1 [file pharmaceuticals-17-01582-s001.zip › pharmaceuticals-3304830-supplementary.pdf]

Supporting information

Study of cytotoxicity of spiro-fused [3-azabicyclo[3.1.0]hexane] oxindoles and cyclopropa[a]pyrrolizidine-oxindoles against tumor cell lines

Anton A. Kornev [1], Stanislav V. Shmakov [1], Alexander I. Ponyaev [2], Alexander V. Stepakov [2,3]* and Vitali M. Boitsov [1,]*

[1] Laboratory of Nanobiotechnologies, Saint-Petersburg National Research Academic University of the Russian Academy of Sciences, Saint Petersburg 194021, Russia
[2] Saint-Petersburg State Institute of Technology, Saint Petersburg 190013, Russia
[3] Department of Chemistry, Saint-Petersburg State University, Saint Petersburg 199034, Russia
* Correspondence: bovitali@yandex.ru (V.M.B.); alstepakov@yandex.ru (A.V.S.)

Table of contents

1. Cytotoxicity of racemic spiro-adducts against K562, HeLa, Jurkat, Sk-mel-2, MCF-7, CT26 cell lines S2
2. Cell distribution over the different phases of the cell cycle S4
3. Copies of ^{1}H NMR spectra of synthesized compounds S4

1. Cytotoxicity of racemic spiro-adducts against K562, HeLa, Jurkat, Sk-mel-2, MCF-7, CT26 cell lines

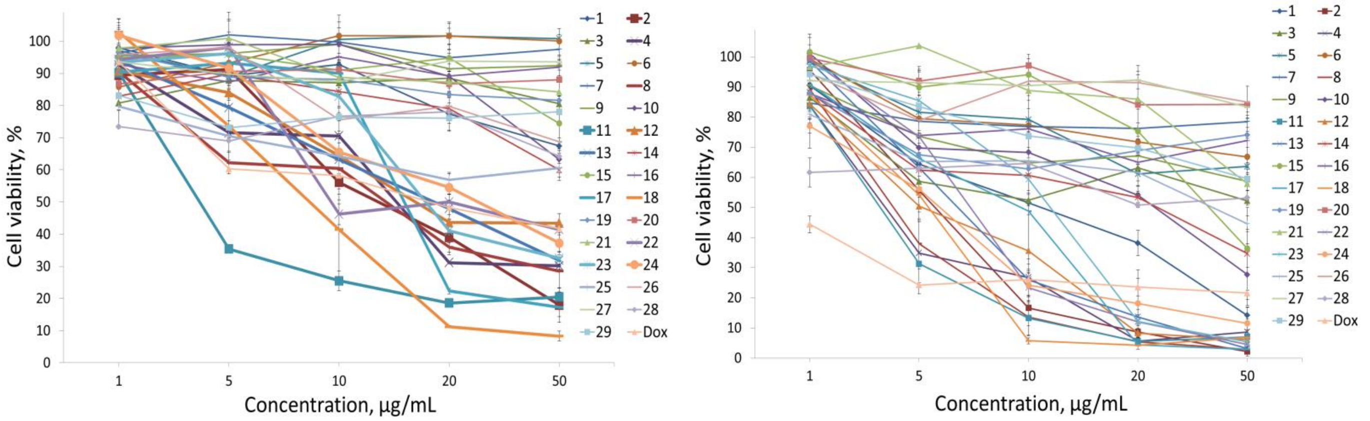

Figure S1. Cytotoxicity of racemic spiro-adducts against the K562 cell line for 24 h (**A**) and 72 h (**B**).

A B

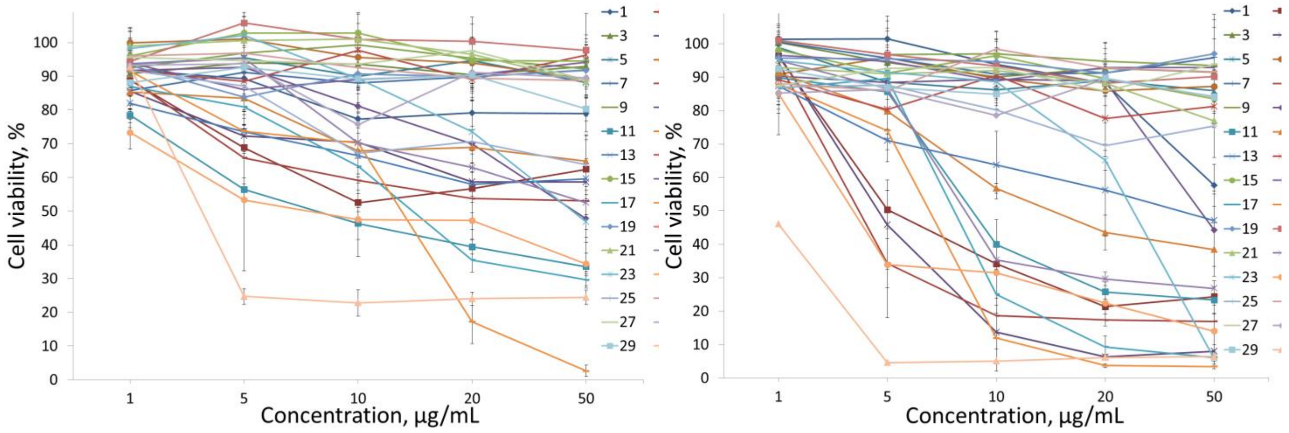

Figure S2. Cytotoxicity of racemic spiro-adducts against the HeLa cell line for 24 h (**A**) and 72 h (**B**).

A B

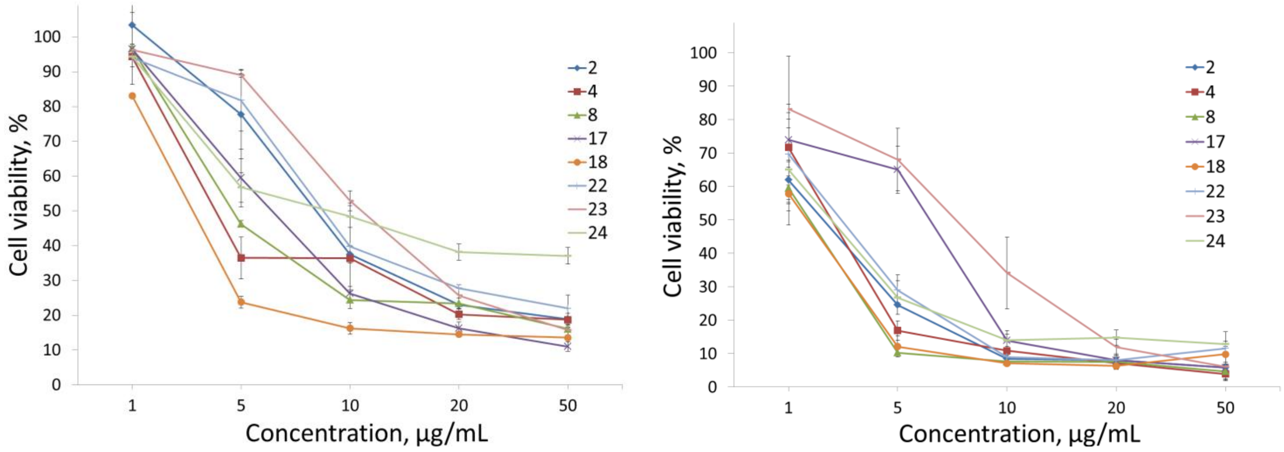

Figure S3. Cytotoxicity of selected racemic spiro-adducts against the Jurkat cell line for 24 h (**A**) and 72 h (**B**).

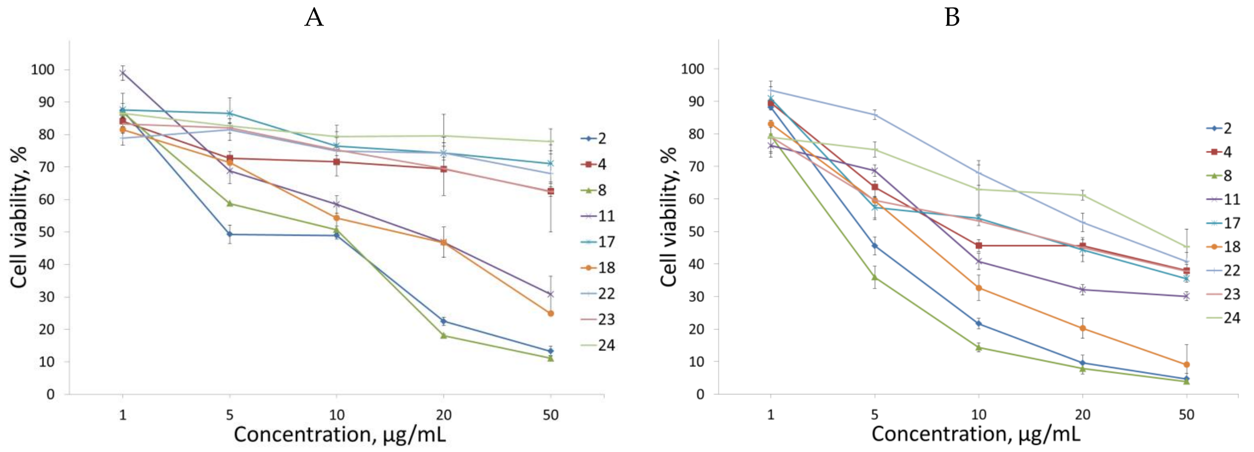

Figure S4. Cytotoxicity of racemic spiro-adducts against the Sk-mel-2 cell line for 24 h (**A**) and 72 h (**B**).

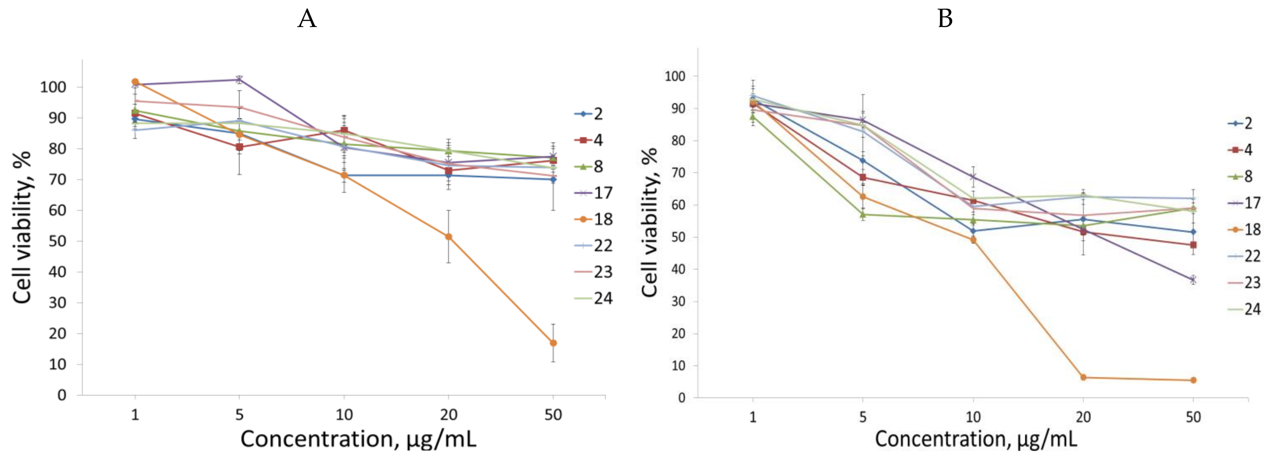

Figure S5. Cytotoxicity of selected racemic spiro-adducts against the MCF-7 cell line for 24 h (**A**) and 72 h (**B**).

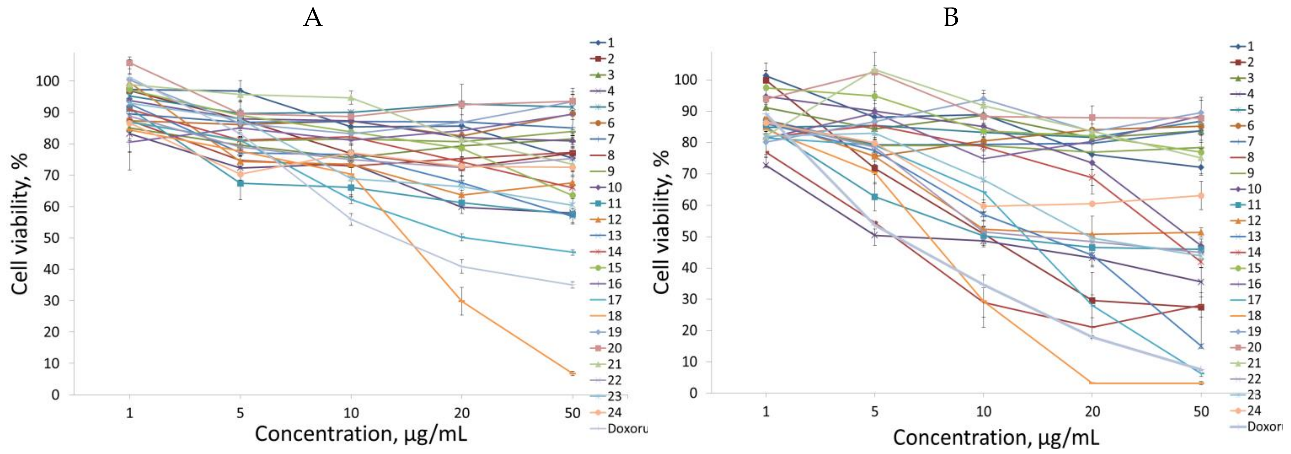

Figure S6. Cytotoxicity of selected racemic spiro-adducts against the CT26 cell line for 24 h (**A**) and 72 h (**B**).

2. Cell distribution over the different phases of the cell cycle

Effect of cycloadducts **2**, **4**, **8**, **17**, **18** at concentrations 5, 10, 20 µg/mL on the distribution of **K562** cells in the cell cycle

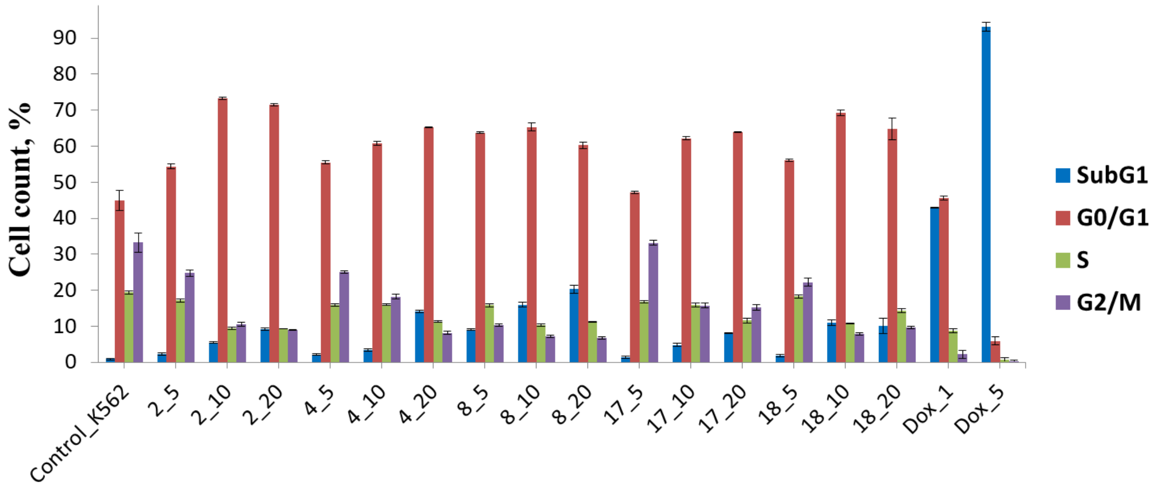

Figure S7. Effect of cycloadducts **2**, **4**, **8**, **17**, **18** at concentrations 5, 10, 20 µg/mL on the distribution of **K562** cells in the cell cycle

3. Copies of ^{1}H NMR spectra of synthesized compounds 1-29

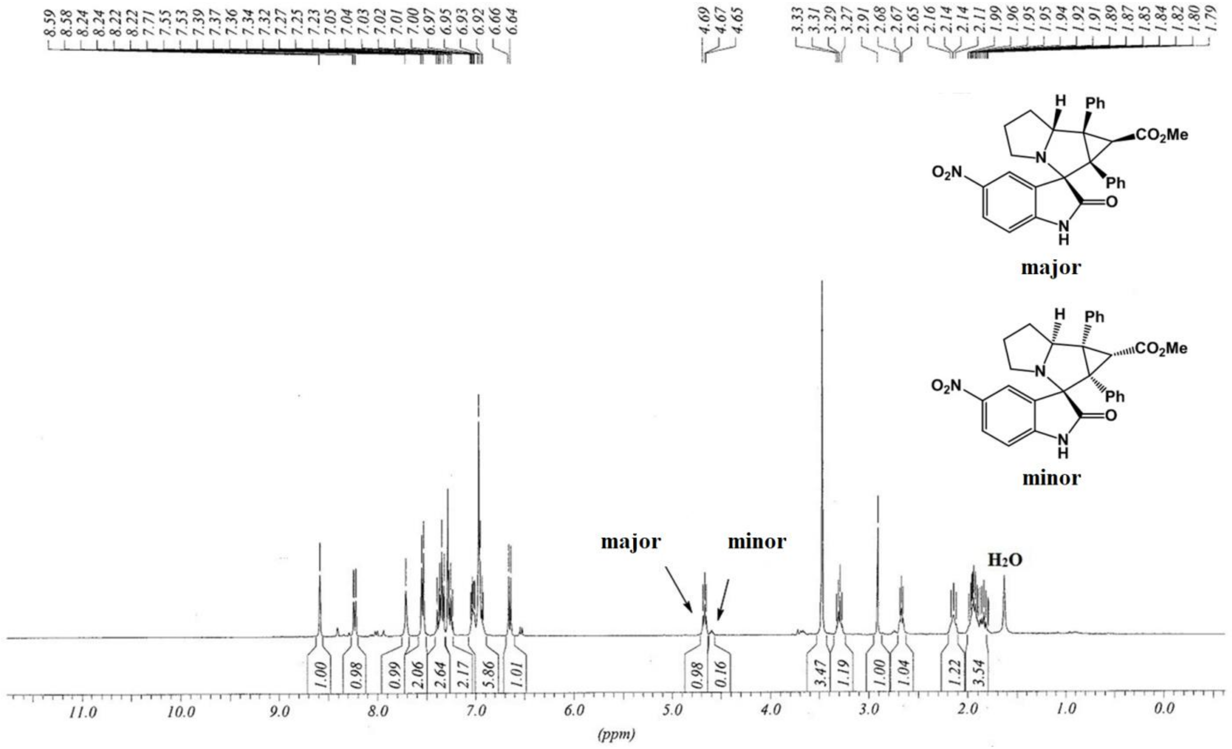

Figure S8. ^{1}H NMR spectrum of inseparable mixture of **1-major** and **1-minor** ($CDCl_3$, 400 MHz).

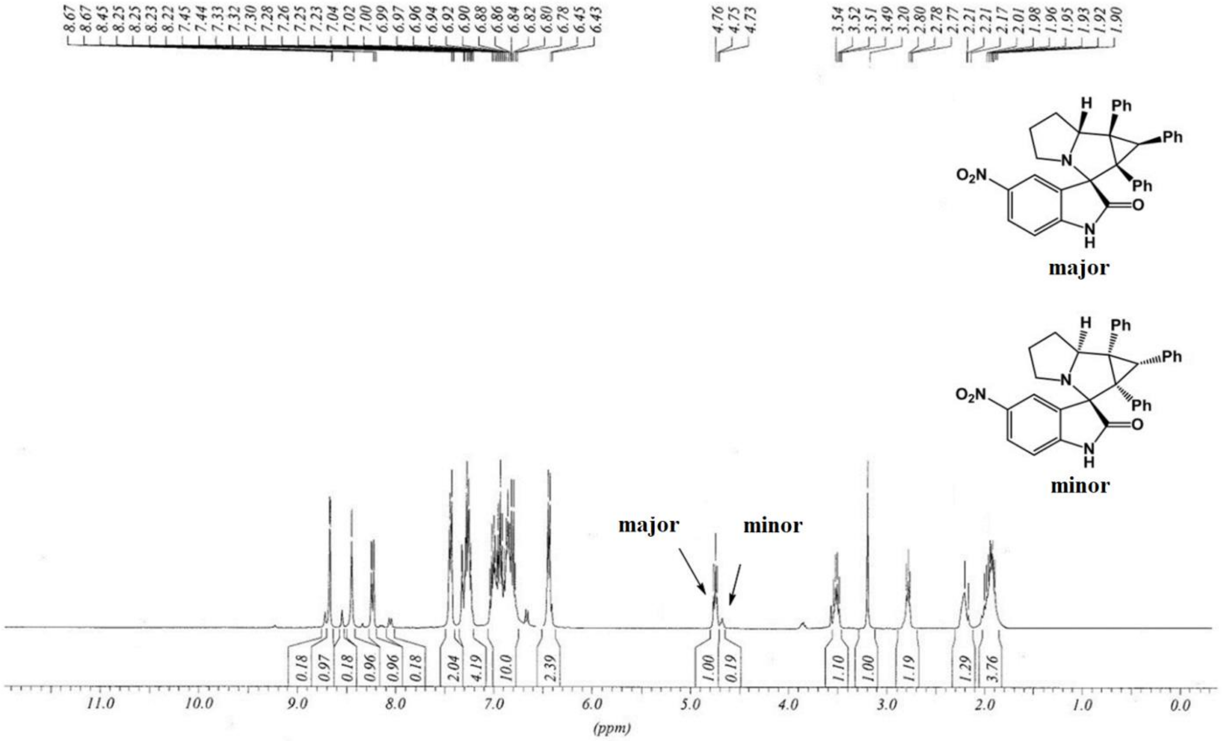

Figure S9. ^{1}H NMR spectrum of inseparable mixture of **2-major** and **2-minor** ($CDCl_3$, 400 MHz).

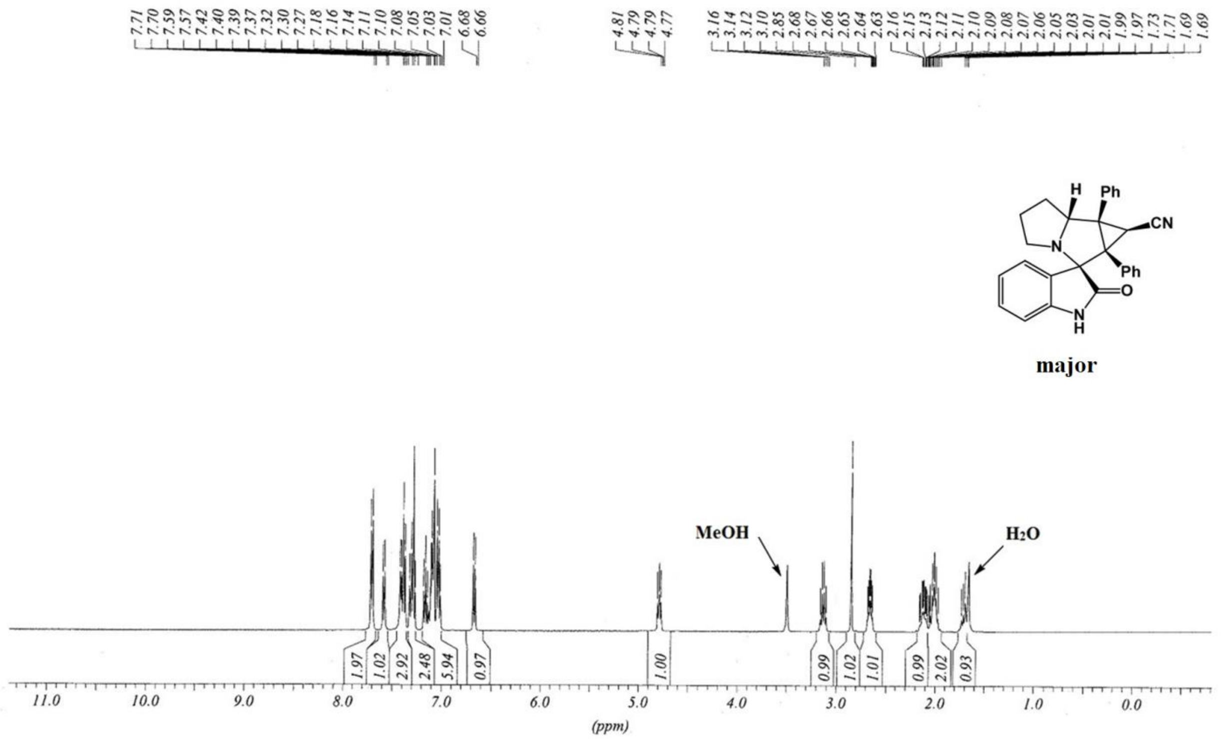

Figure S10. ^{1}H NMR spectrum of compound **3-major** ($CDCl_3$, 400 MHz).

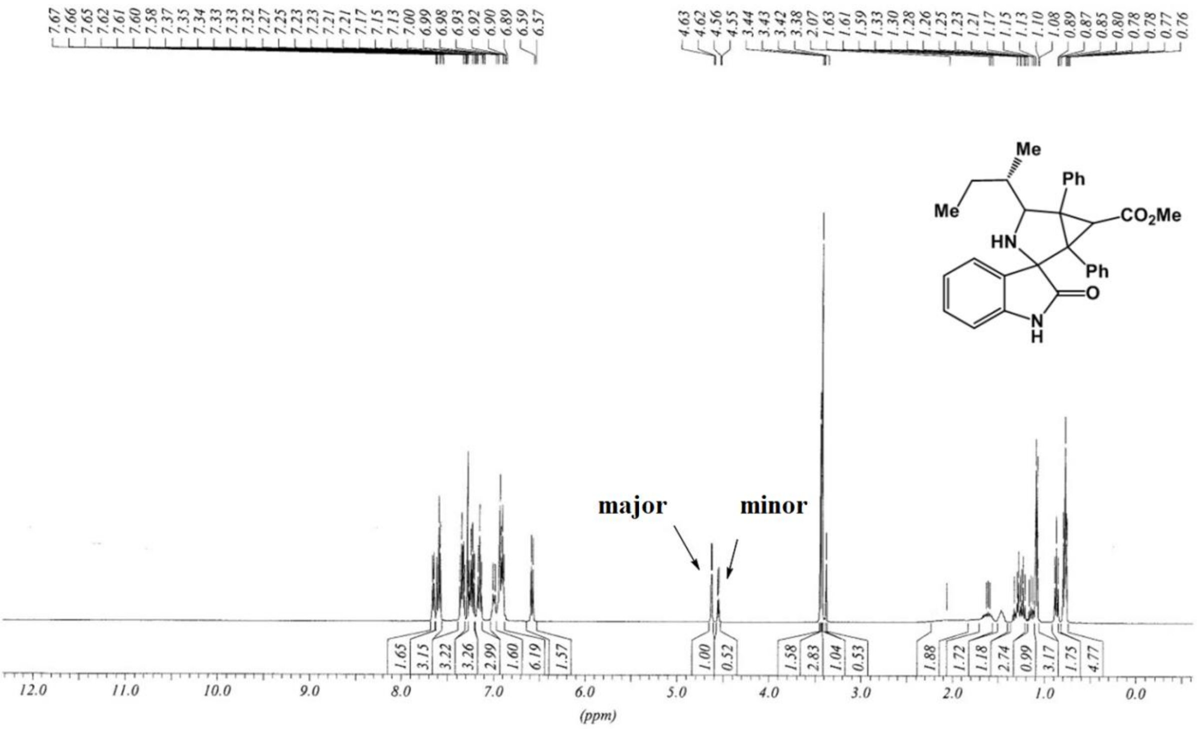

Figure S11. ^{1}H NMR spectrum of inseparable mixture of **4-major** and **4-minor** ($CDCl_3$, 400 MHz).

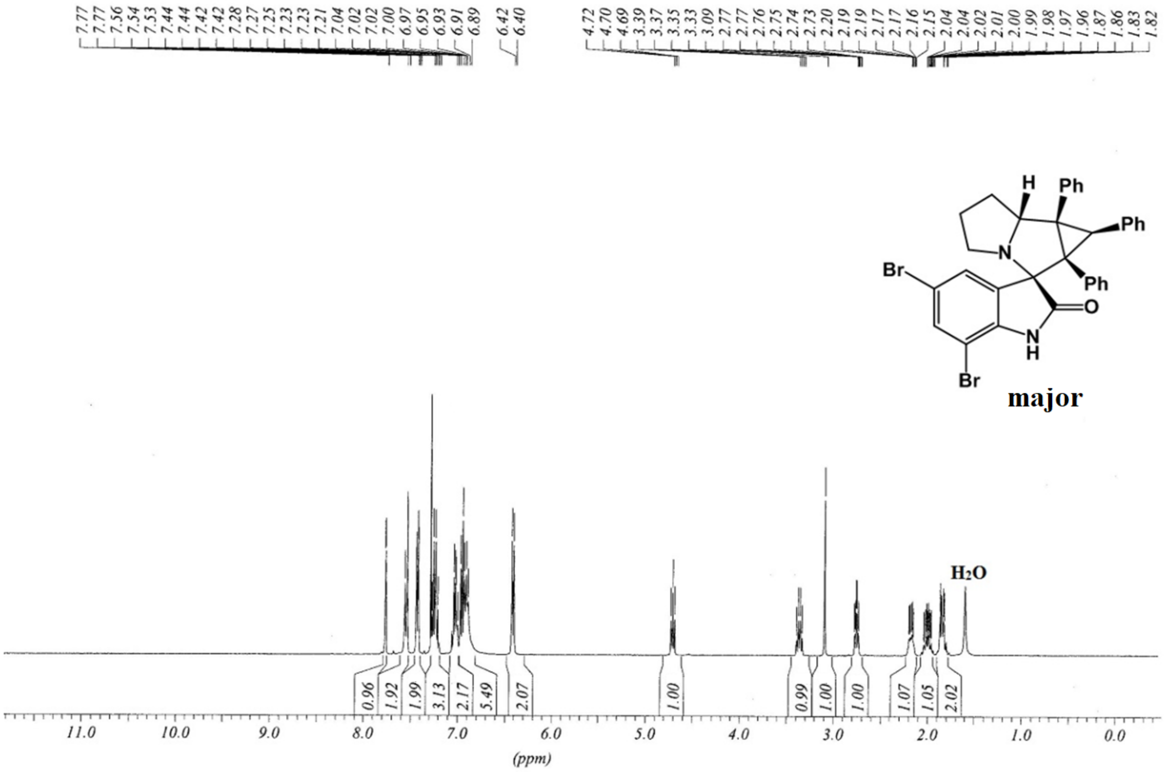

Figure S12. ^{1}H NMR spectrum of compound **5-major** ($CDCl_3$, 400 MHz).

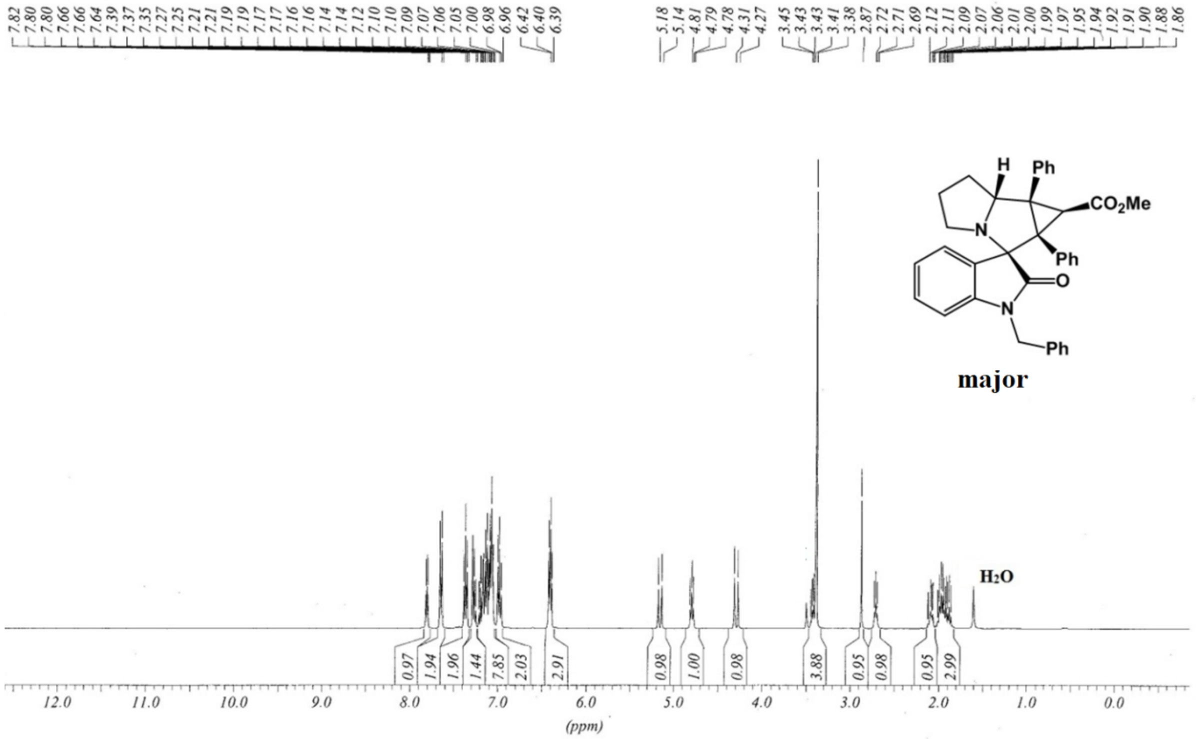

Figure S13. ^{1}H NMR spectrum of compound **6-major** ($CDCl_3$, 400 MHz).

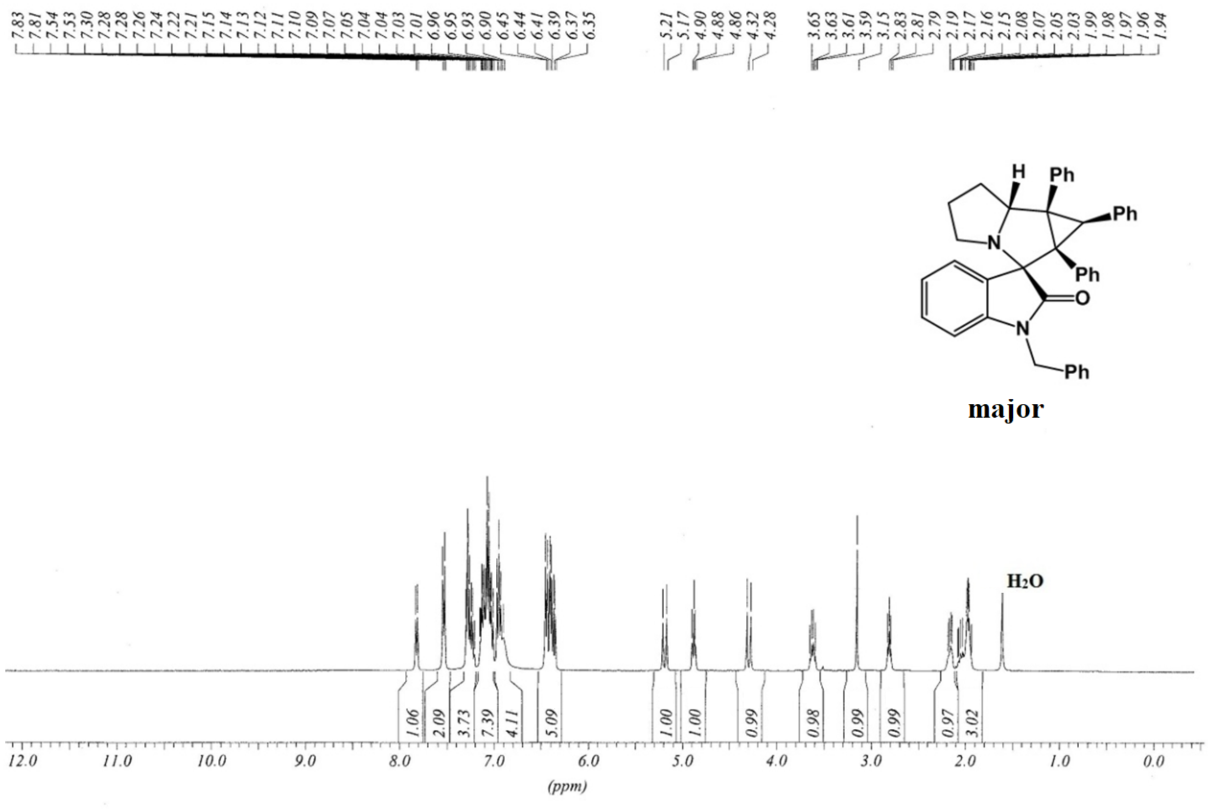

Figure S14. ^{1}H NMR spectrum of compound **7-major** ($CDCl_3$, 400 MHz).

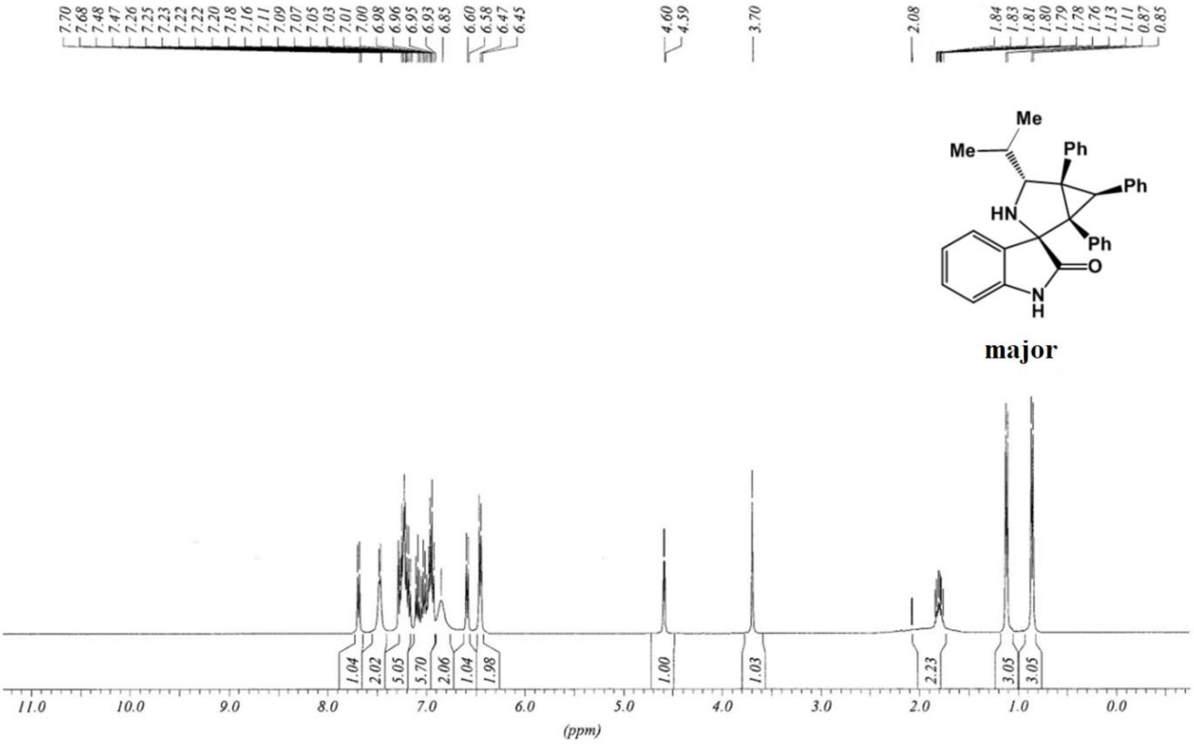

Figure S15. ^{1}H NMR spectrum of compound **8-major** ($CDCl_3$, 400 MHz).

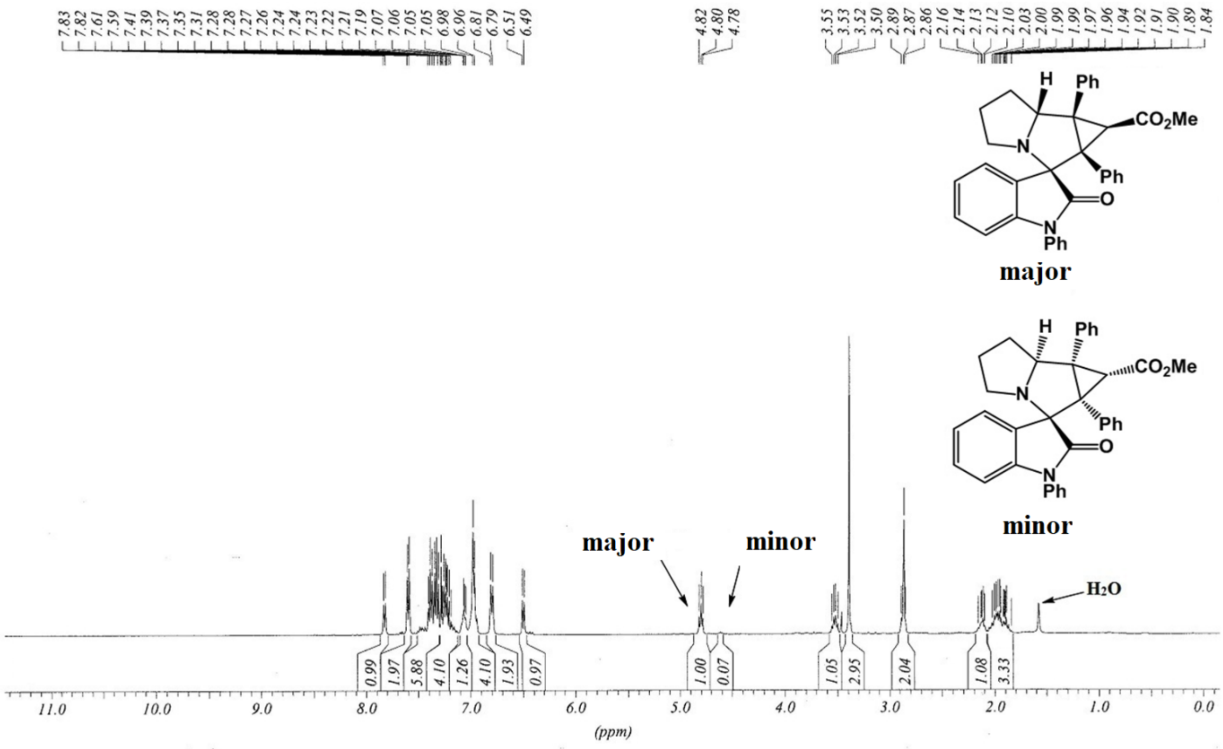

Figure S16. ^{1}H NMR spectrum of inseparable mixture of **9-major** and **9-minor** ($CDCl_3$, 400 MHz).

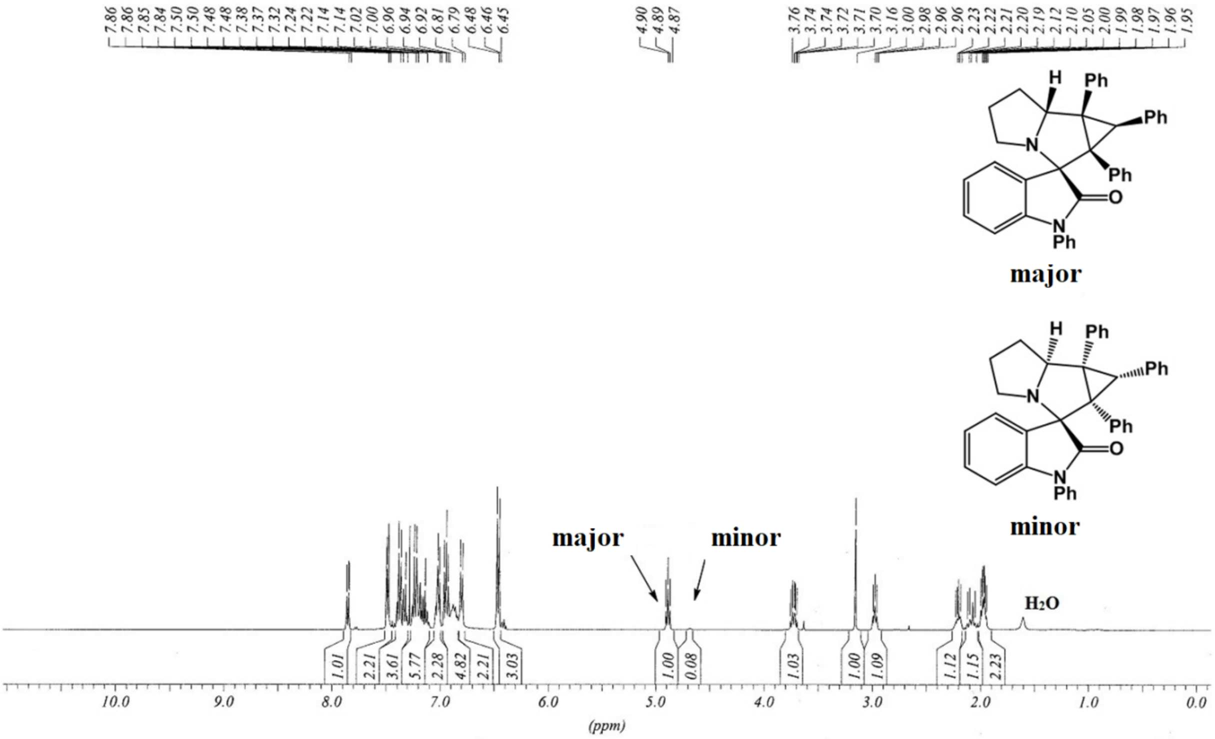

Figure S17. ^{1}H NMR spectrum of inseparable mixture of **10-major** and **10-minor** ($CDCl_3$, 400 MHz).

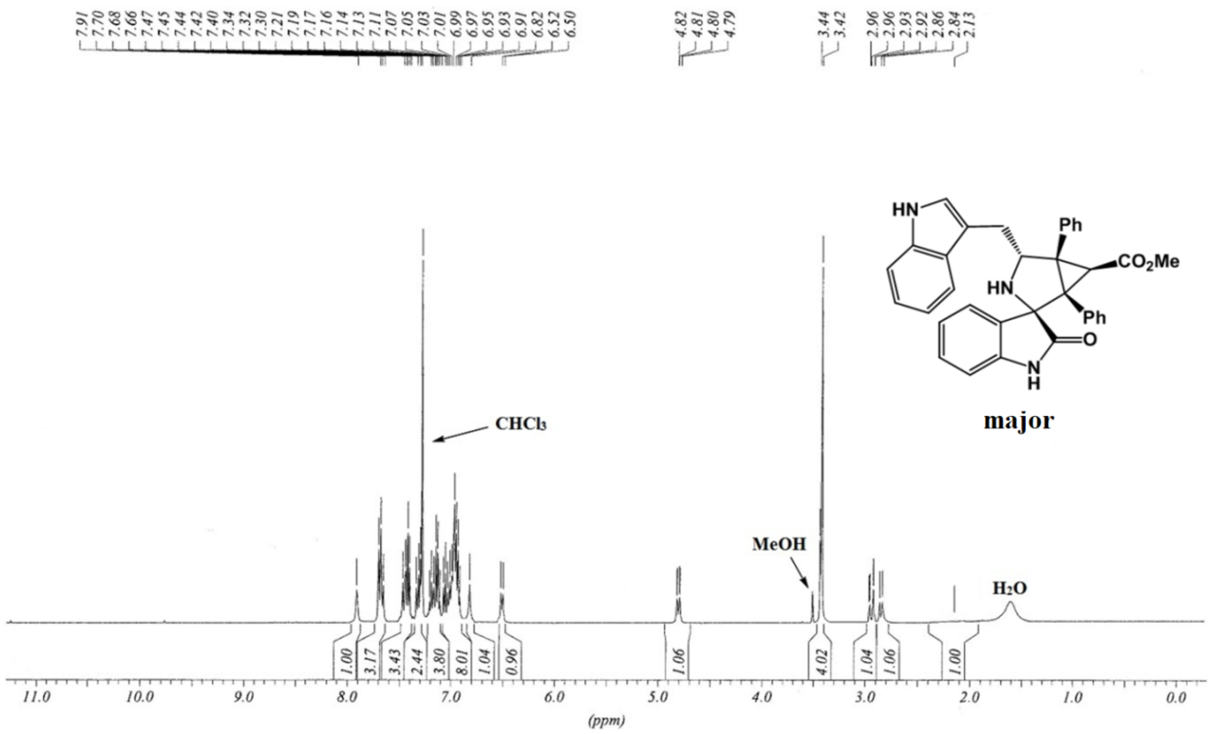

Figure S18. ^{1}H NMR spectrum of compound **11-major** ($CDCl_3$, 400 MHz).

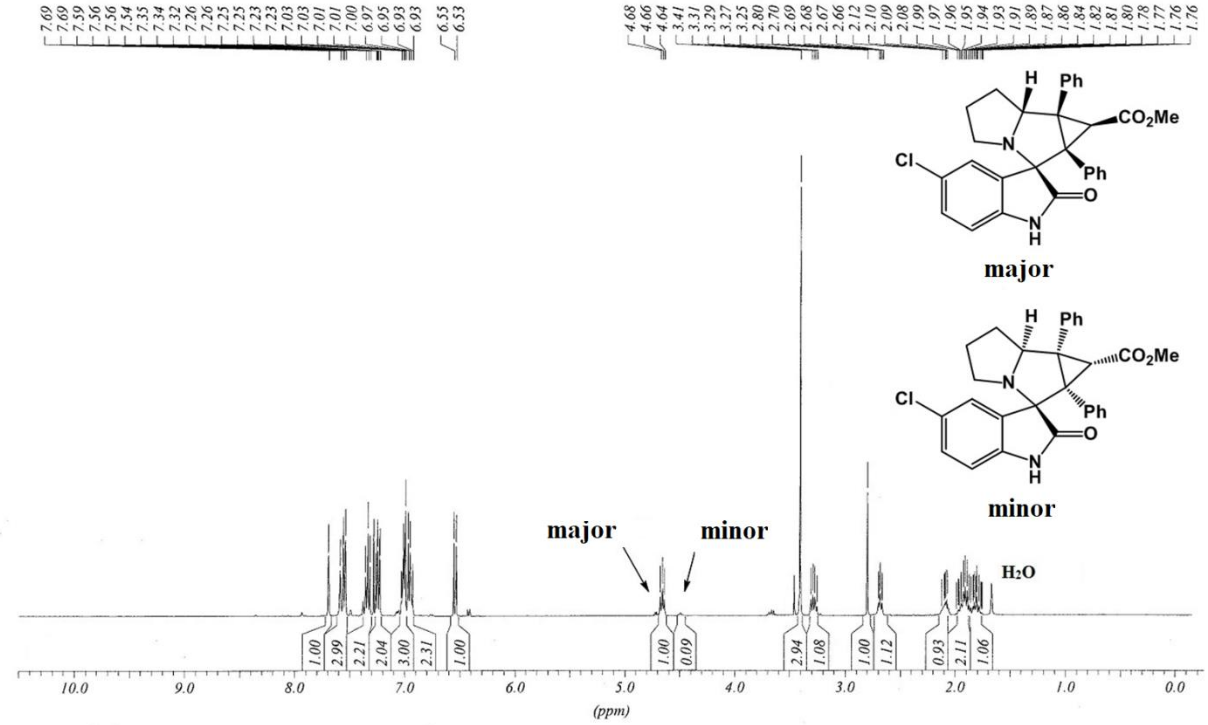

Figure S19. ^{1}H NMR spectrum of inseparable mixture of **12-major** and **12-minor** ($CDCl_3$, 400 MHz).

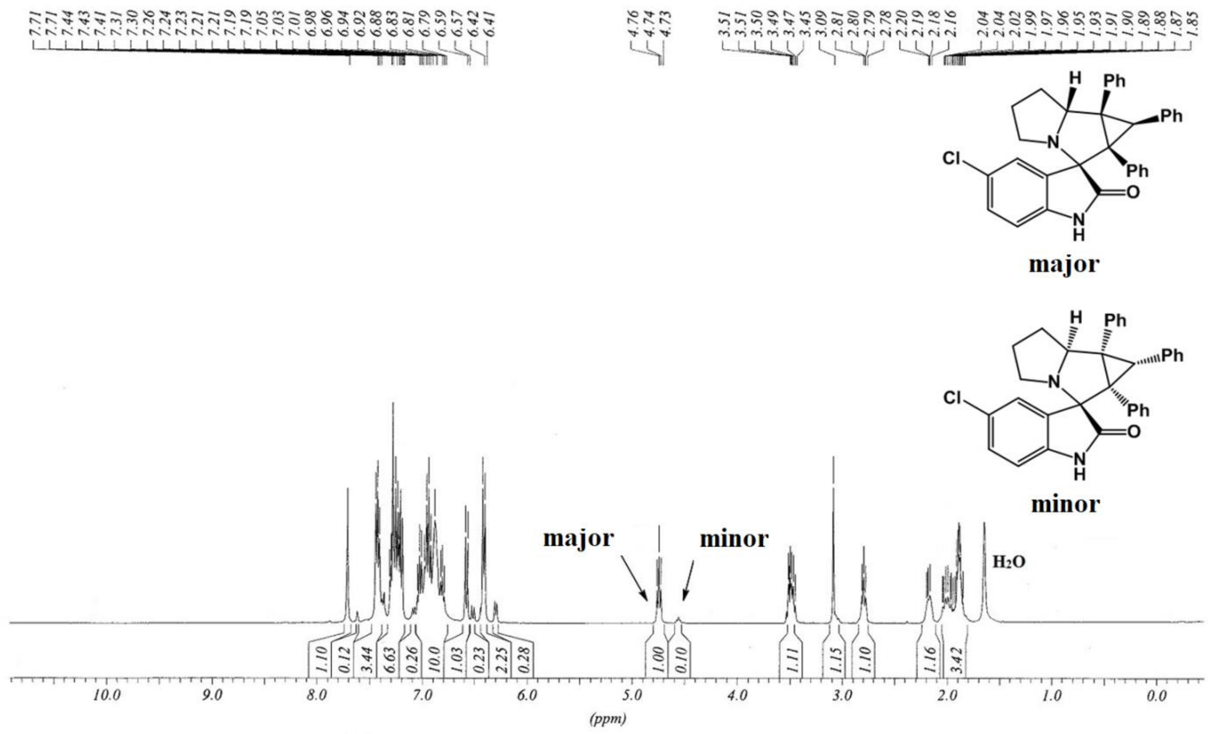

Figure S20. ^{1}H NMR spectrum of inseparable mixture of **13-major** and **13-minor** ($CDCl_3$, 400 MHz).

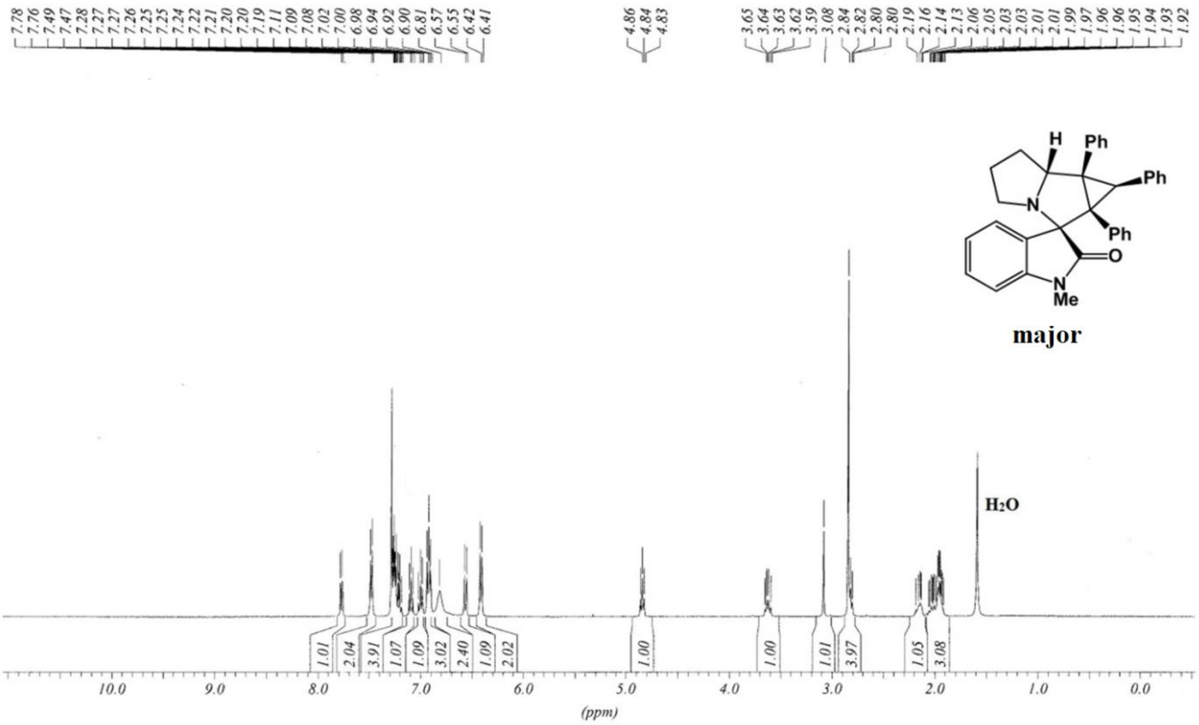

Figure S21. ^{1}H NMR spectrum of compound **14-major** ($CDCl_3$, 400 MHz).

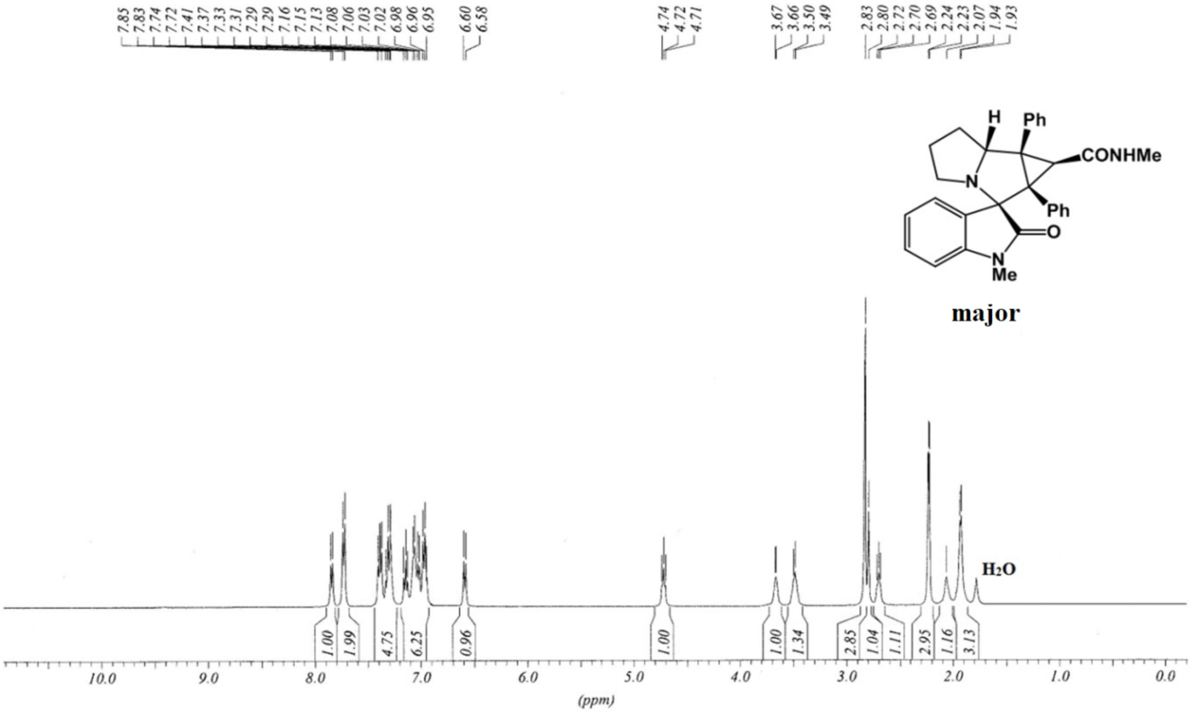

Figure S22. ^{1}H NMR spectrum of compound **15-major** ($CDCl_3$, 400 MHz).

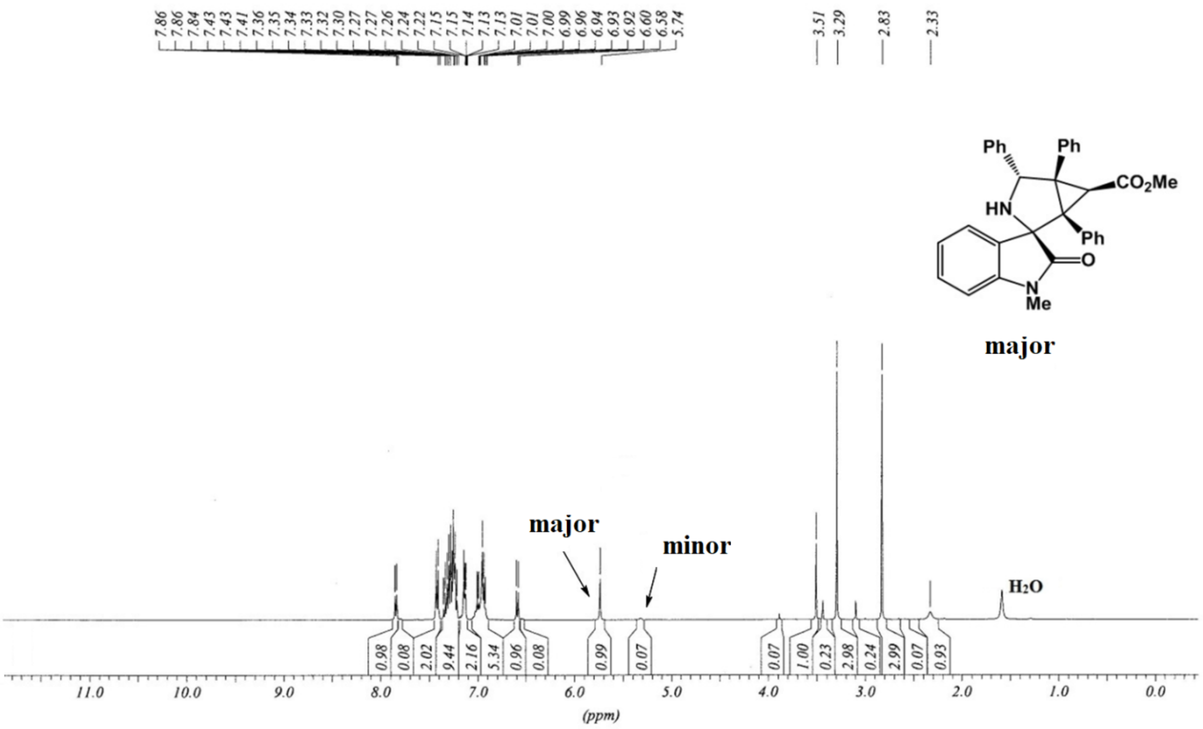

Figure S23. ^{1}H NMR spectrum of inseparable mixture of **16-major** and **16-minor** ($CDCl_3$, 400 MHz).

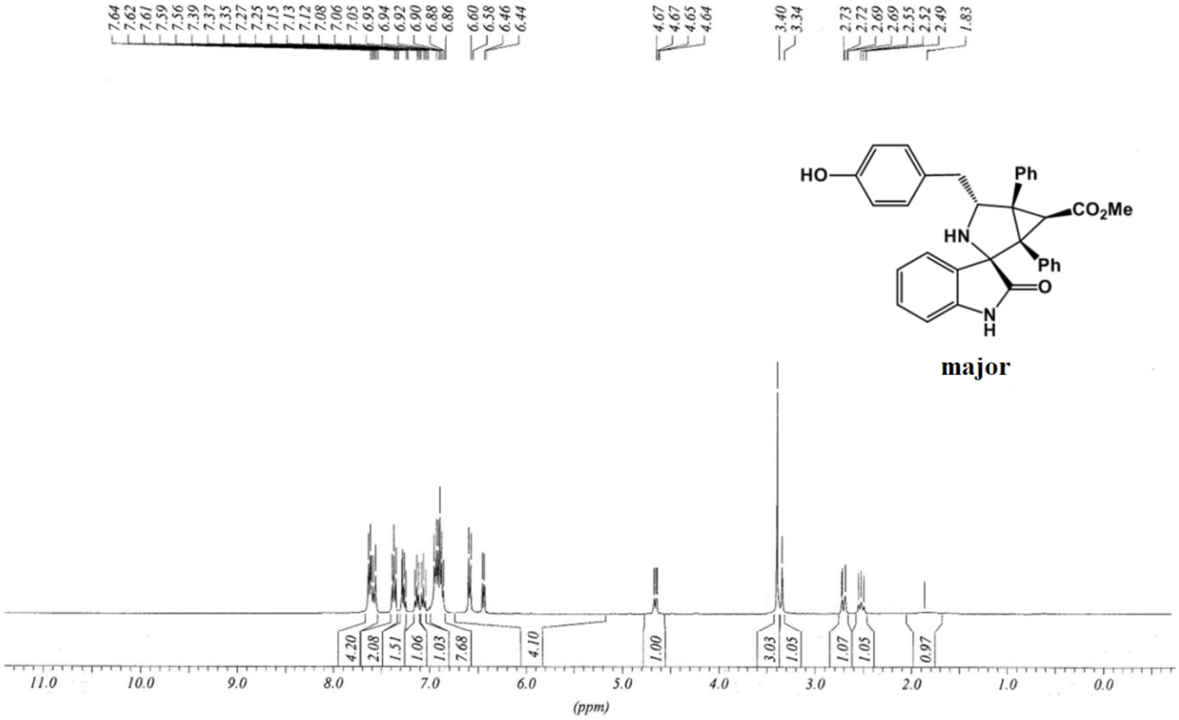

Figure S24. ^{1}H NMR spectrum of compound **17-major** ($CDCl_3$, 400 MHz).

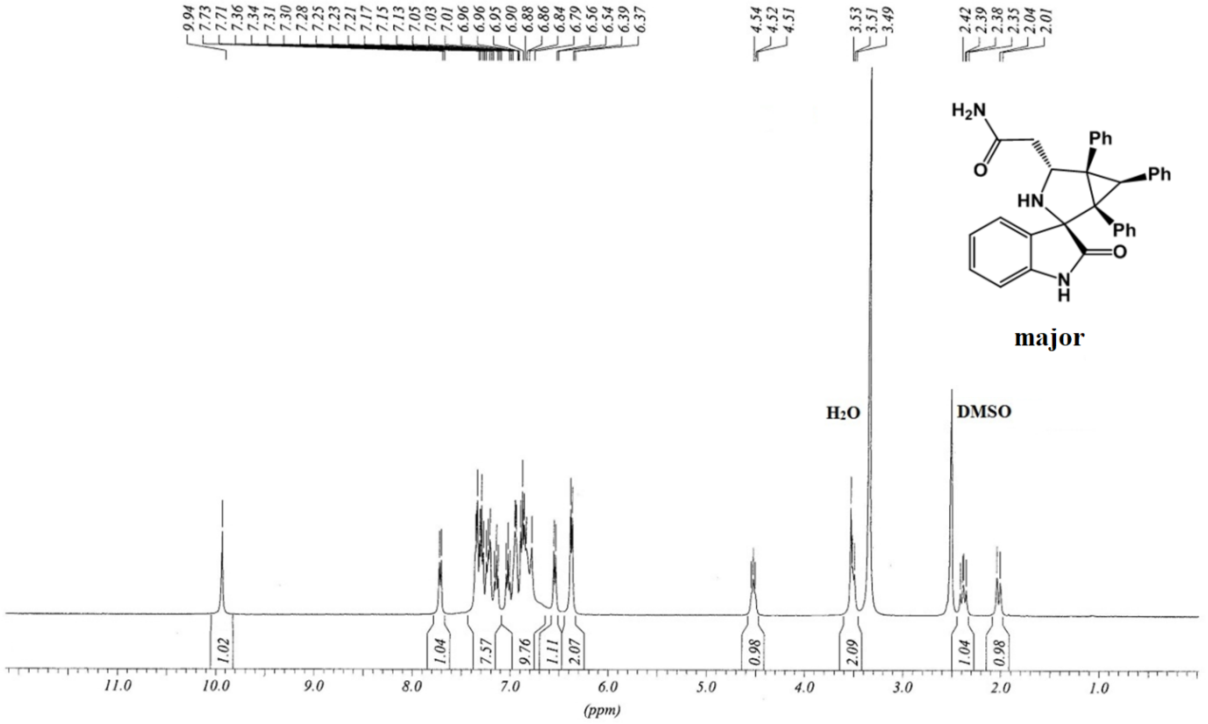

Figure S25. ^{1}H NMR spectrum of compound **18-major** (DMSO-d_6, 400 MHz).

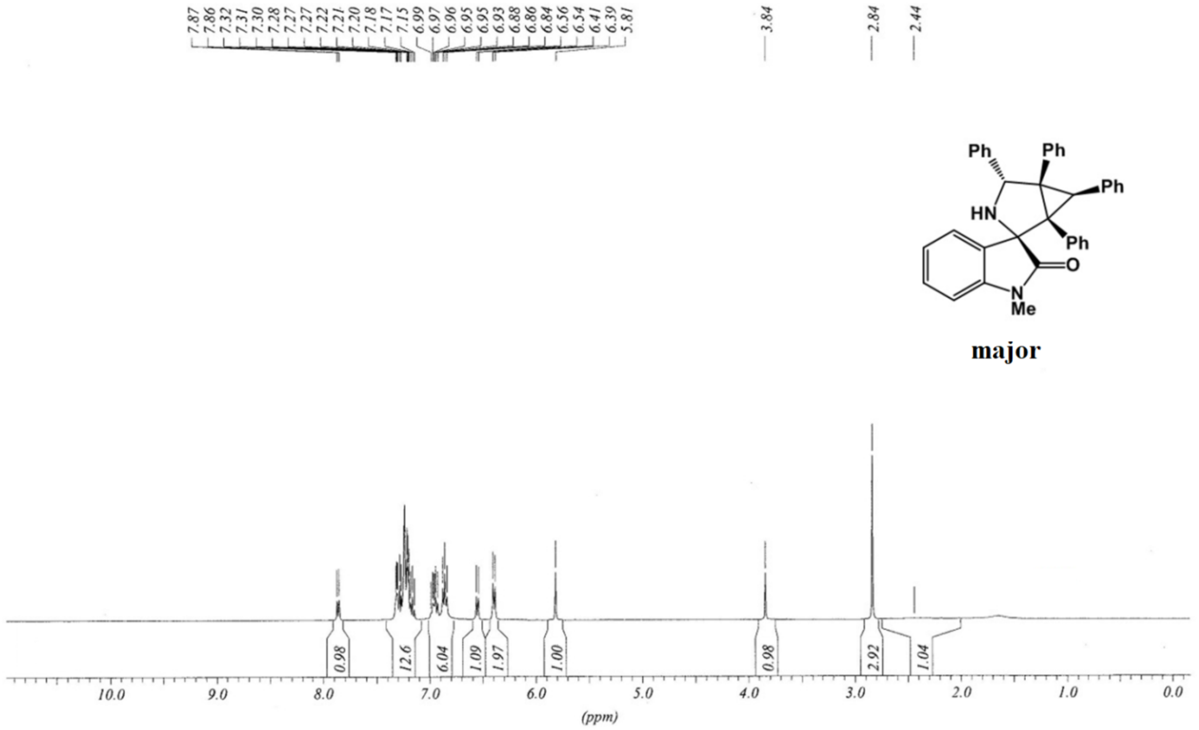

Figure S26. ^{1}H NMR spectrum of compound **19-major** ($CDCl_3$, 400 MHz).

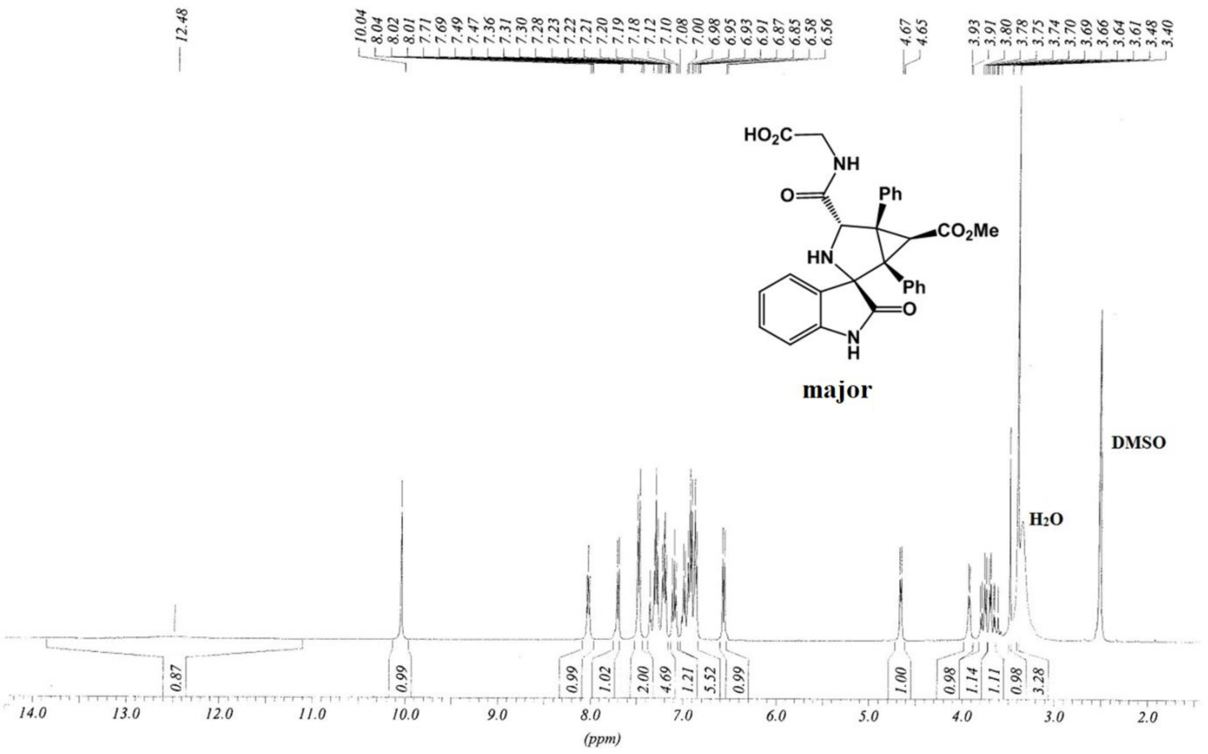

Figure S27. ^{1}H NMR spectrum of compound **20-major** ($CDCl_3$, 400 MHz).

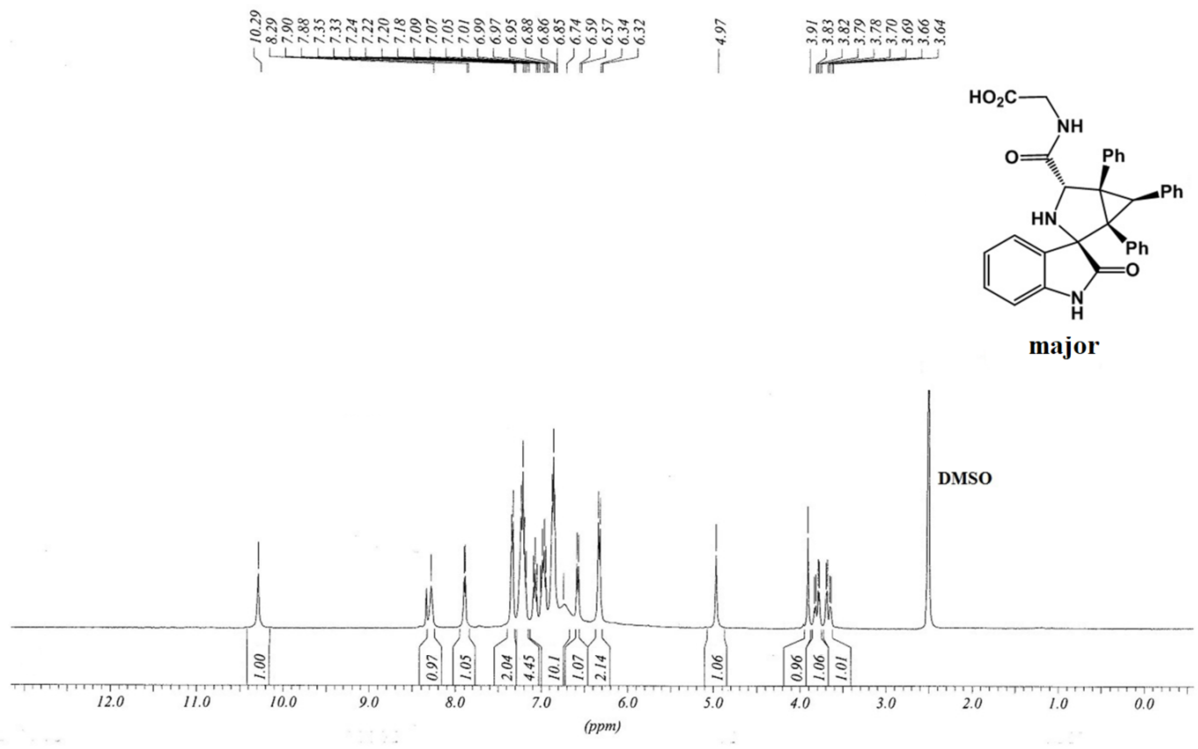

Figure S28. ^{1}H NMR spectrum of compound **21-major** ($CDCl_3$, 400 MHz).

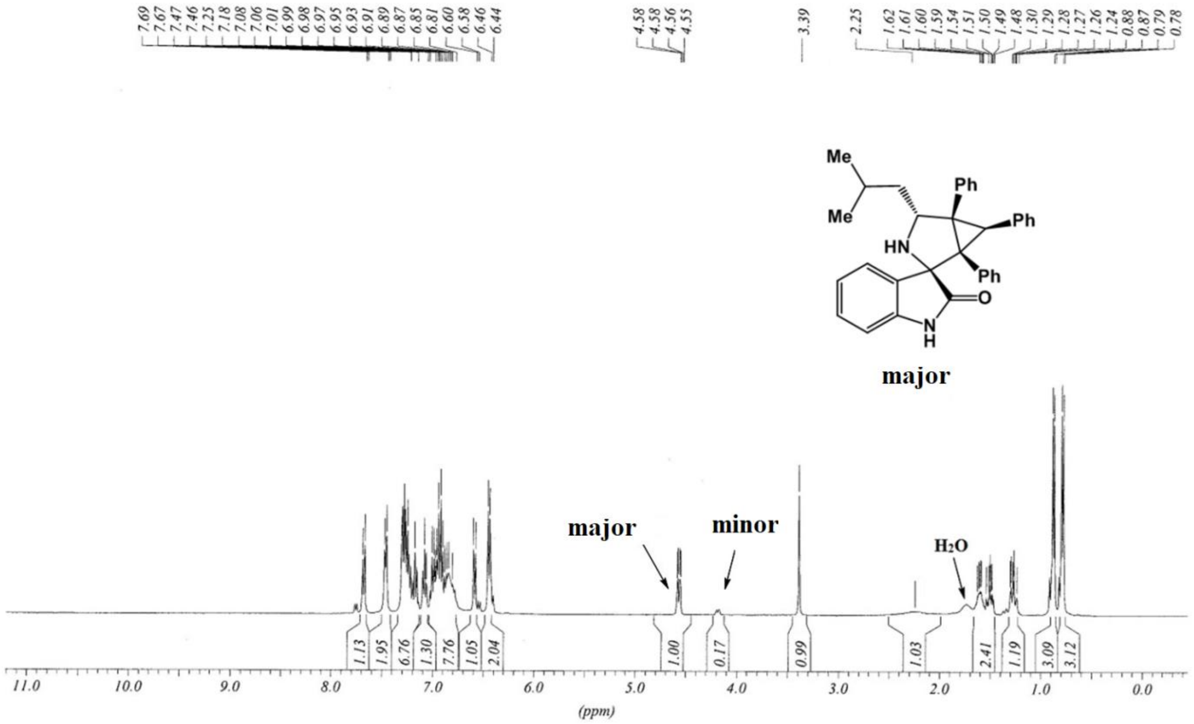

Figure S29. ^{1}H NMR spectrum of inseparable mixture of **22-major** and **22-minor** ($CDCl_3$, 400 MHz).

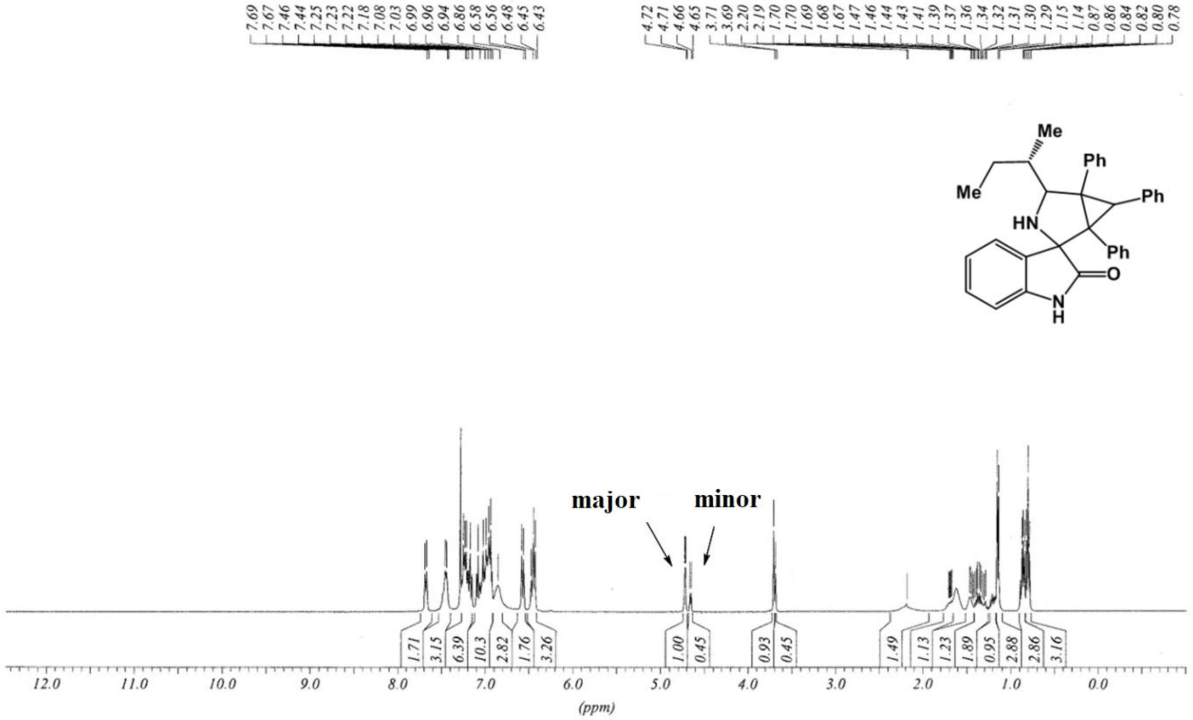

Figure S30. ^{1}H NMR spectrum of inseparable mixture of **23-major** and **23-minor** ($CDCl_3$, 400 MHz).

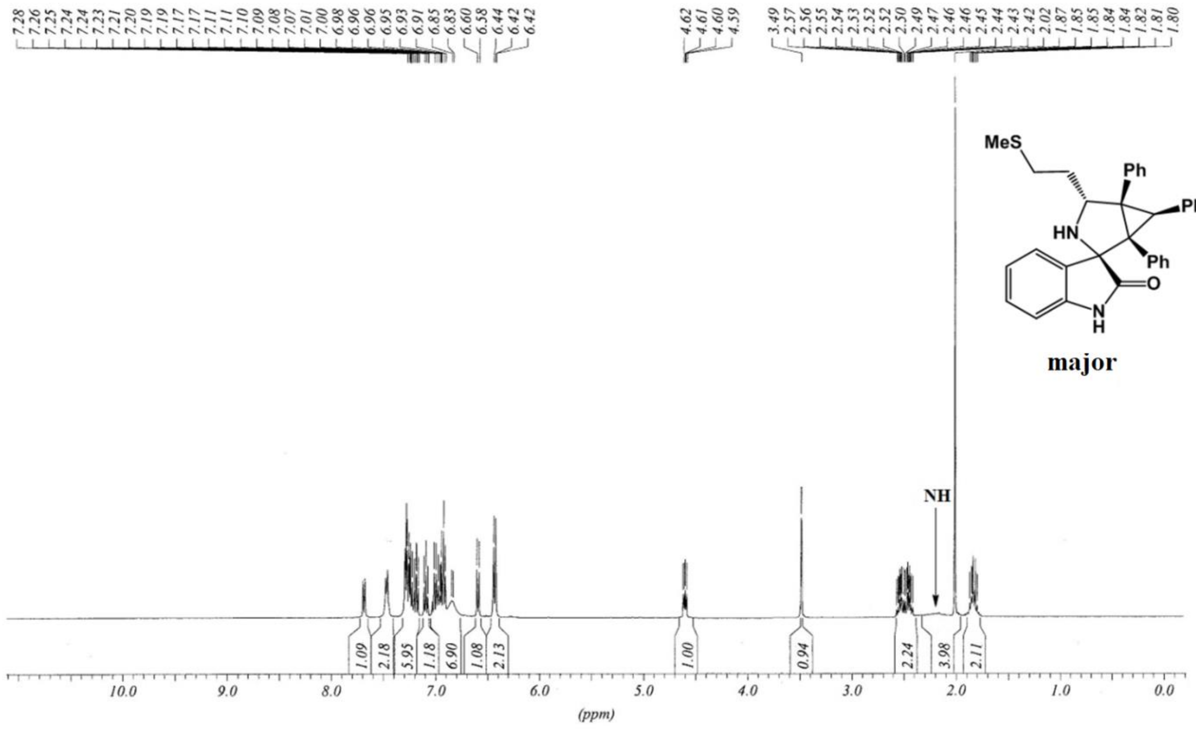

Figure S31. ^{1}H NMR spectrum of compound **24-major** ($CDCl_3$, 400 MHz).

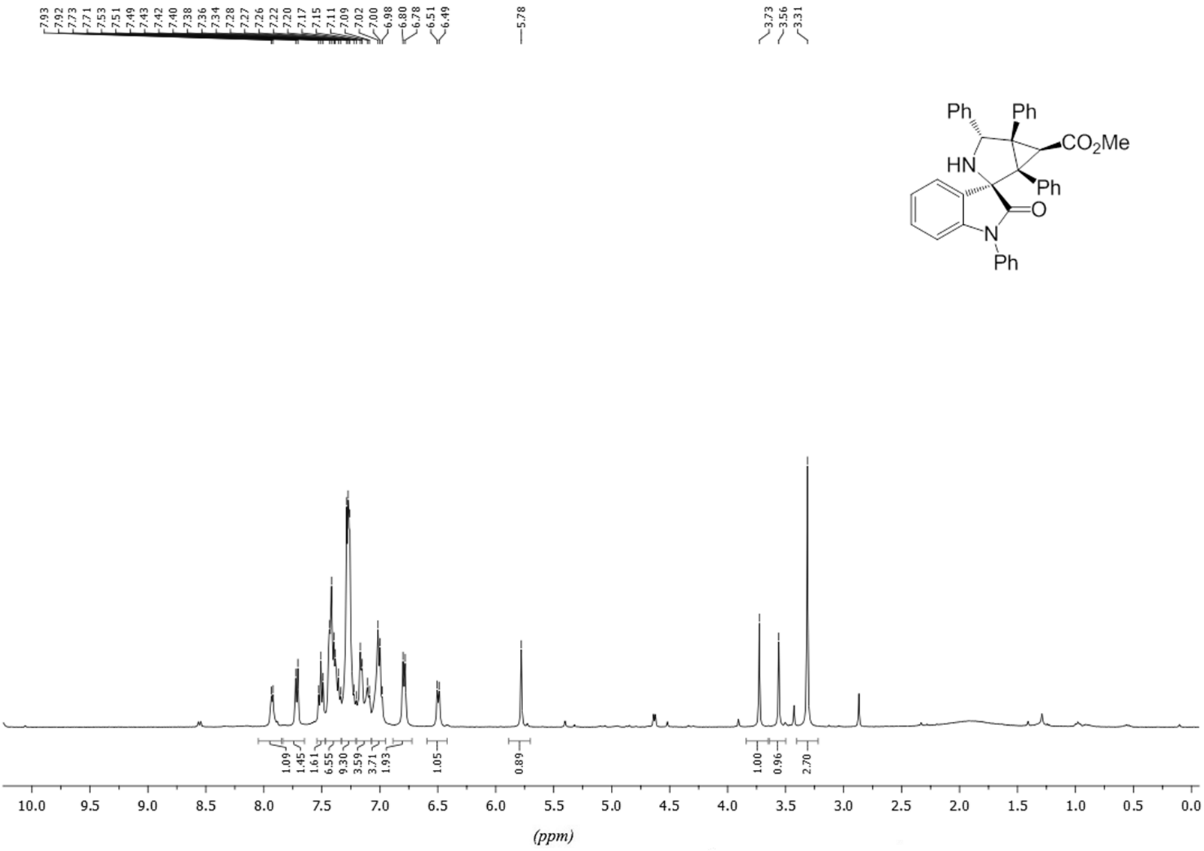

Figure S32. ^{1}H NMR spectrum of inseparable mixture of **25-major** and **25-minor** ($CDCl_3$, 400 MHz).

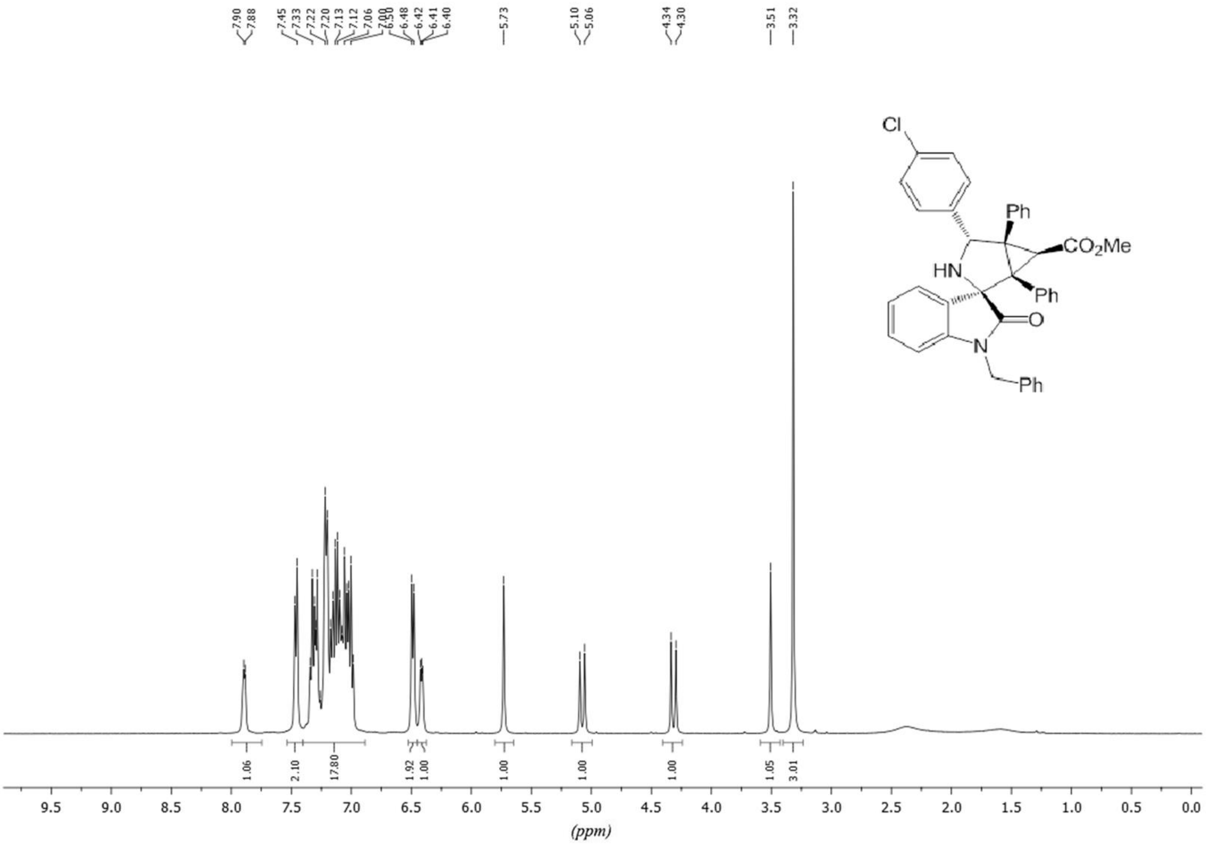

Figure S33. ^{1}H NMR spectrum of compound **26-major** ($CDCl_3$, 400 MHz).

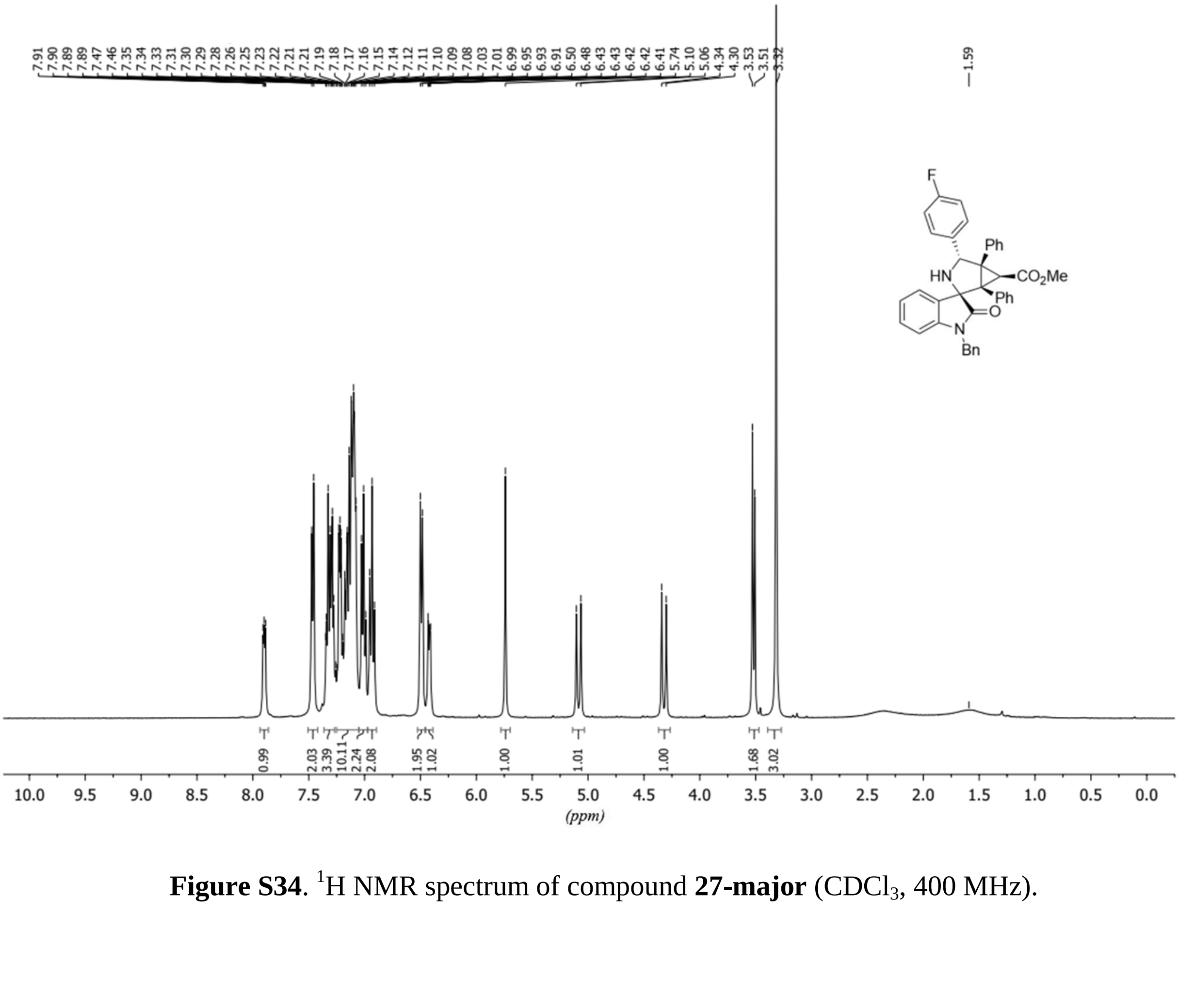

Figure S34. ^{1}H NMR spectrum of compound **27-major** ($CDCl_3$, 400 MHz).

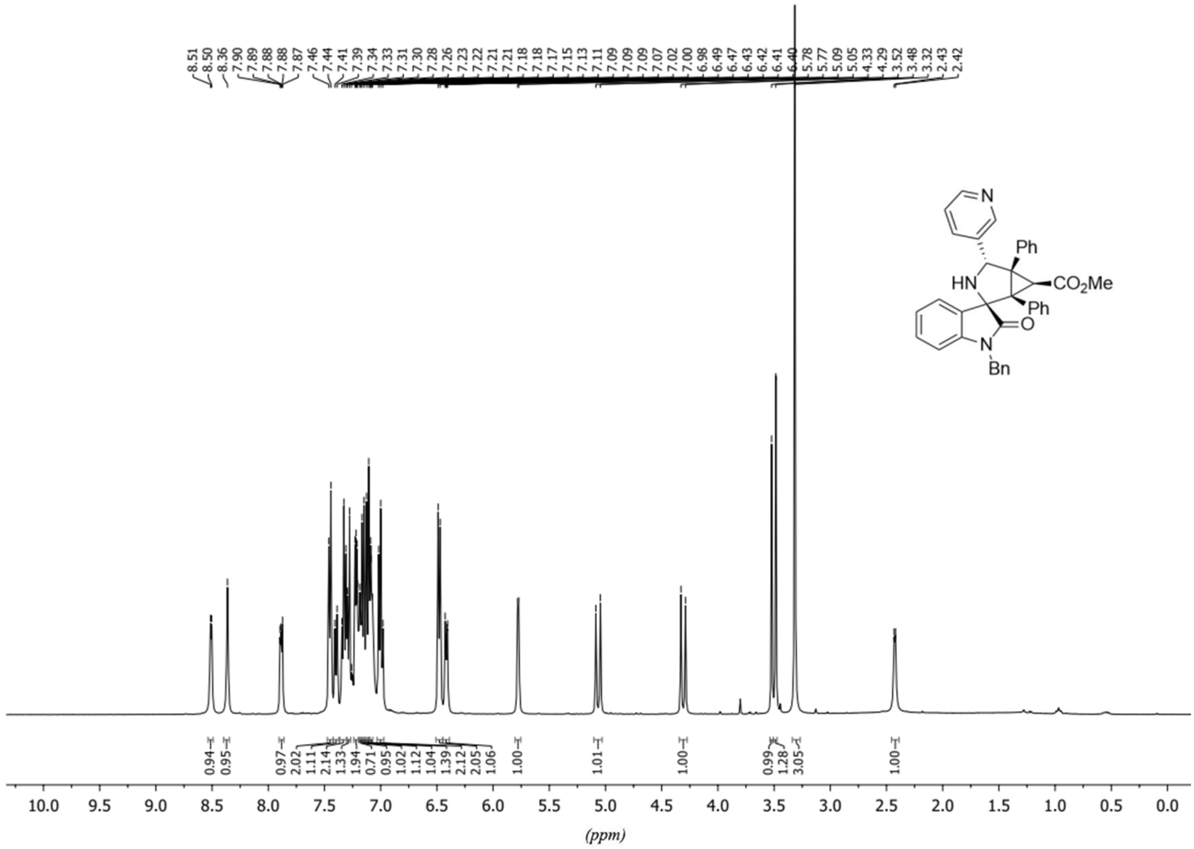

Figure S35. ^{1}H NMR spectrum of compound **28-major** ($CDCl_3$, 400 MHz).

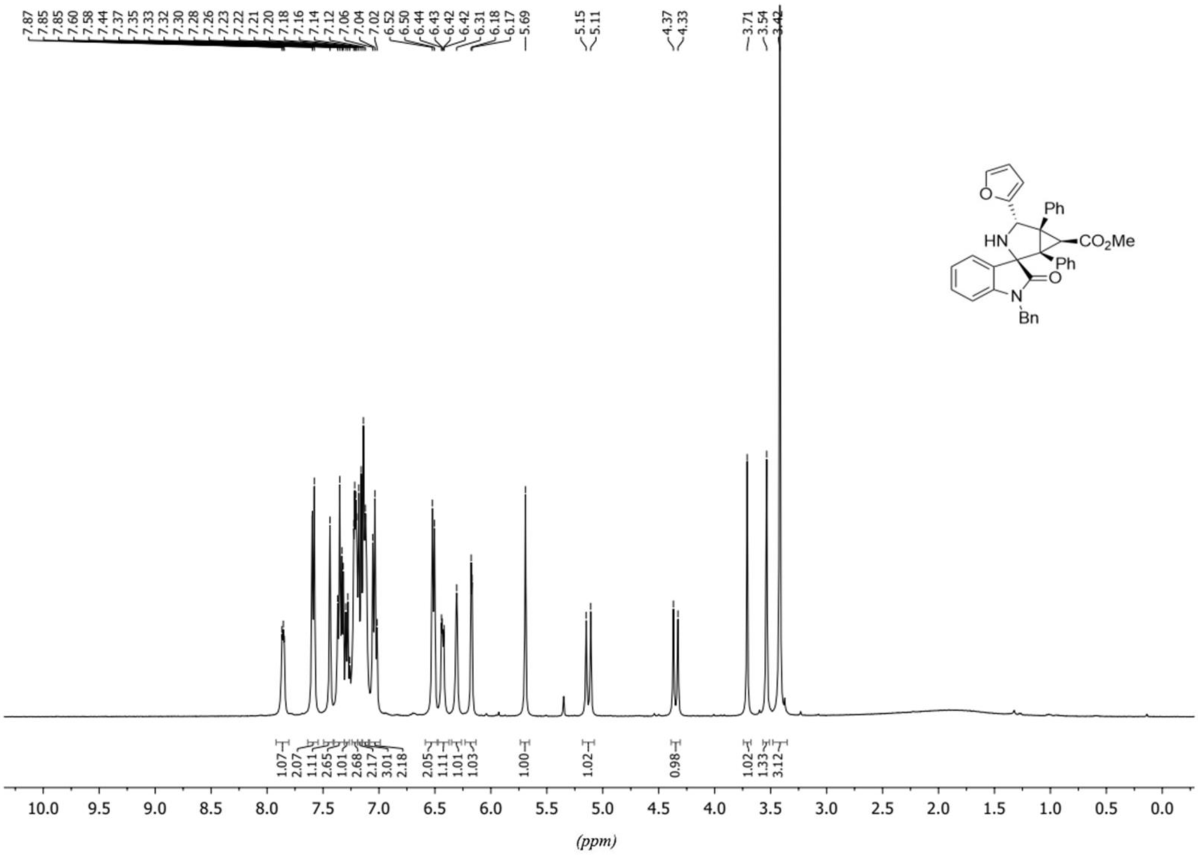

Figure S36. ^{1}H NMR spectrum of compound **29-major** ($CDCl_3$, 400 MHz).
